# Targeted sequencing reveals expanded genetic diversity of human transfer RNAs

**DOI:** 10.1080/15476286.2019.1646079

**Published:** 2019-08-13

**Authors:** Matthew D. Berg, Daniel J. Giguere, Jacqueline S. Dron, Jeremy T. Lant, Julie Genereaux, Calwing Liao, Jian Wang, John F. Robinson, Gregory B. Gloor, Robert A. Hegele, Patrick O’Donoghue, Christopher J. Brandl

**Affiliations:** a Department of Biochemistry, The University of Western Ontario, London, ON, Canada; b Robarts Research Institute, The University of Western Ontario, London, ON, Canada; c Department of Medicine, The University of Western Ontario, London, ON, Canada; d Department of Chemistry, The University of Western Ontario, London, ON, Canada

**Keywords:** tRNA biology, human tRNA variation, tRNA-encoding genes, tRNA capture panel, translation

## Abstract

Transfer RNAs are required to translate genetic information into proteins as well as regulate other cellular processes. Nucleotide changes in tRNAs can result in loss or gain of function that impact the composition and fidelity of the proteome. Despite links between tRNA variation and disease, the importance of cytoplasmic tRNA variation has been overlooked. Using a custom capture panel, we sequenced 605 human tRNA-encoding genes from 84 individuals. We developed a bioinformatic pipeline that allows more accurate tRNA read mapping and identifies multiple polymorphisms occurring within the same variant. Our analysis identified 522 unique tRNA-encoding sequences that differed from the reference genome from 84 individuals. Each individual had ~66 tRNA variants including nine variants found in less than 5% of our sample group. Variants were identified throughout the tRNA structure with 17% predicted to enhance function. Eighteen anticodon mutants were identified including potentially mistranslating tRNAs; e.g., a tRNA^Ser^ that decodes Phe codons. Similar engineered tRNA variants were previously shown to inhibit cell growth, increase apoptosis and induce the unfolded protein response in mammalian cell cultures and chick embryos. Our analysis shows that human tRNA variation has been underestimated. We conclude that the large number of tRNA genes provides a buffer enabling the emergence of variants, some of which could contribute to disease.

## Introduction

**Figure 1.**
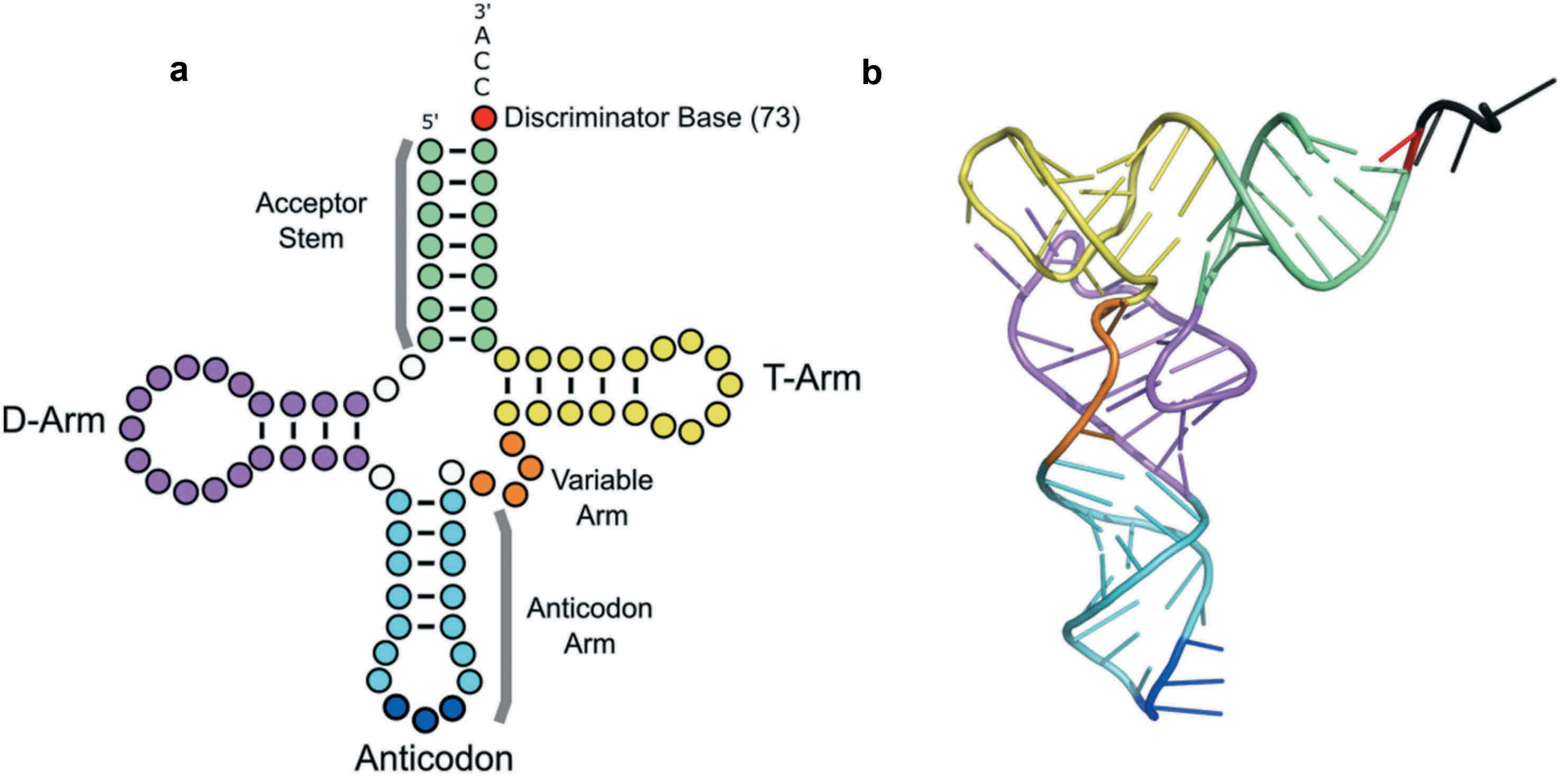
tRNA structure. (a) tRNAs are represented as cloverleaf structures in two dimensions. (b) In three dimensions, tRNAs fold into an L-shape stabilized by intramolecular base-pairing shown here by the tRNA^Phe^ structure [PDB: 1HEZ; [20]]. In both diagrams, the tRNA structural elements are colored: acceptor stem (green), dihydrouridine (D)-arm (purple), anticodon stem (light blue), anticodon (bases 34, 35, 36 in dark blue), variable arm (orange), T-arm (yellow) and the discriminator base (red).

tRNAs play a fundamental role in the cell by contributing an essential activity to protein synthesis [1] as well as functioning as non-coding RNAs in an expanding number of cellular regulatory networks [reviewed in [2]]. In translation, tRNAs are substrates for aminoacyl-tRNA synthetases that ligate amino acids onto their cognate tRNAs in an ATP dependent reaction [reviewed in [3]]. The ribosome and EF-Tu (eEF1α in eukaryotes) must recognize and accommodate all families of the aminoacyl-tRNA isoacceptors, thus constraining the sequence and shape of tRNAs over evolutionary time [4–11]. In two-dimensions, the 74–93 nucleotides of different tRNAs are represented as a cloverleaf structure with four or five double-stranded stems resulting from internal base-pairing ([12], Figure 1a). The 3ʹ-N_73_CCA_76_ end is aminoacylated at A76 by the cognate aminoacyl-tRNA synthetase [reviewed in [13]]. The discriminator (nucleotide 73), a major determinant for charging for many tRNAs [14,15], is followed by the universally conserved 3ʹ-CCA, which is added post-transcriptionally in eukaryotes [16,17]. Other conserved features are the dihydrouridine (D) arm, anticodon stem-loop and the ribothymidine (T) arm [18,19]. In three dimensions, tRNAs fold into a common L-shaped structure [20-22], (Figure 1b). The acceptor stem stacks on top of the T-arm to form the shorter branch of the L-shape, and the long branch of the L-shape consists of the anticodon stem interacting with the D-arm. In tRNA^Ser^, tRNA^Leu^ and tRNA^Sec^, an additional variable arm is situated 3ʹ of the anticodon stem.

Due to their complex molecular interactions, single nucleotide changes in tRNAs can result in loss or gain of function that impact the composition and fidelity of the proteome. Mutations of both types have already been linked to disease in mice and humans [23–26]. For example, a mutation in a brain-specific tRNA^Arg^ gene causes neurodegeneration in mice when combined with a second mutation in *GTPBP2*, a gene involved in ribosome recycling [27]. In another case, a serine tRNA that mistranslates serine at alanine codons promotes cellular transformation, stimulates angiogenesis and produces faster growing tumors compared to controls in mice [28]. In humans, a single nucleotide mutation in tRNA^Sec^ causes fatigue, muscle weakness, abdominal pain, thyroid dysfunction and low selenium levels [29].

Despite links between tRNA variation and disease, the importance of tRNA variation has often been over-looked. In part, this is due to the extensive base modification of tRNAs [reviewed in [30]] and the resulting difficulty in their direct sequencing using standard protocols [31]. Variation can be analyzed at the genome level; however, the similarity of tRNA sequences requires that whole-genome sequencing be at sufficient depth to recognize differences amongst as many as 25 closely related genes and that sufficient flanking sequence be included to accurately map a variant to a specific tRNA gene. Furthermore, many of the genome-wide association studies for human disease are limited to exome capture and sequencing, missing the tRNA-encoding genes and other non-coding RNA genes entirely.

To more accurately estimate the extent of tRNA variation in the human population, we created a custom tRNA gene capture panel to sequence the 610 human tRNA-encoding genes. The problems of mapping similar tRNAs to unique loci and identifying multiple polymorphisms within the same allele were dealt with by maximizing read depth and selecting reads that span entire tRNA genes including locus specific flanking sequence. Whereas the 1000 Genomes Project suggests that individuals carry one or two variants on average [32], our analysis uncovered far greater natural human tRNA variation. We conclude that the large number of human tRNA genes provides a natural buffer enabling the emergence of tRNA variants, including those that affect function with the potential to contribute to disease.

## Results

### Targeted sequencing of human tRNA genes

**Figure 2.**
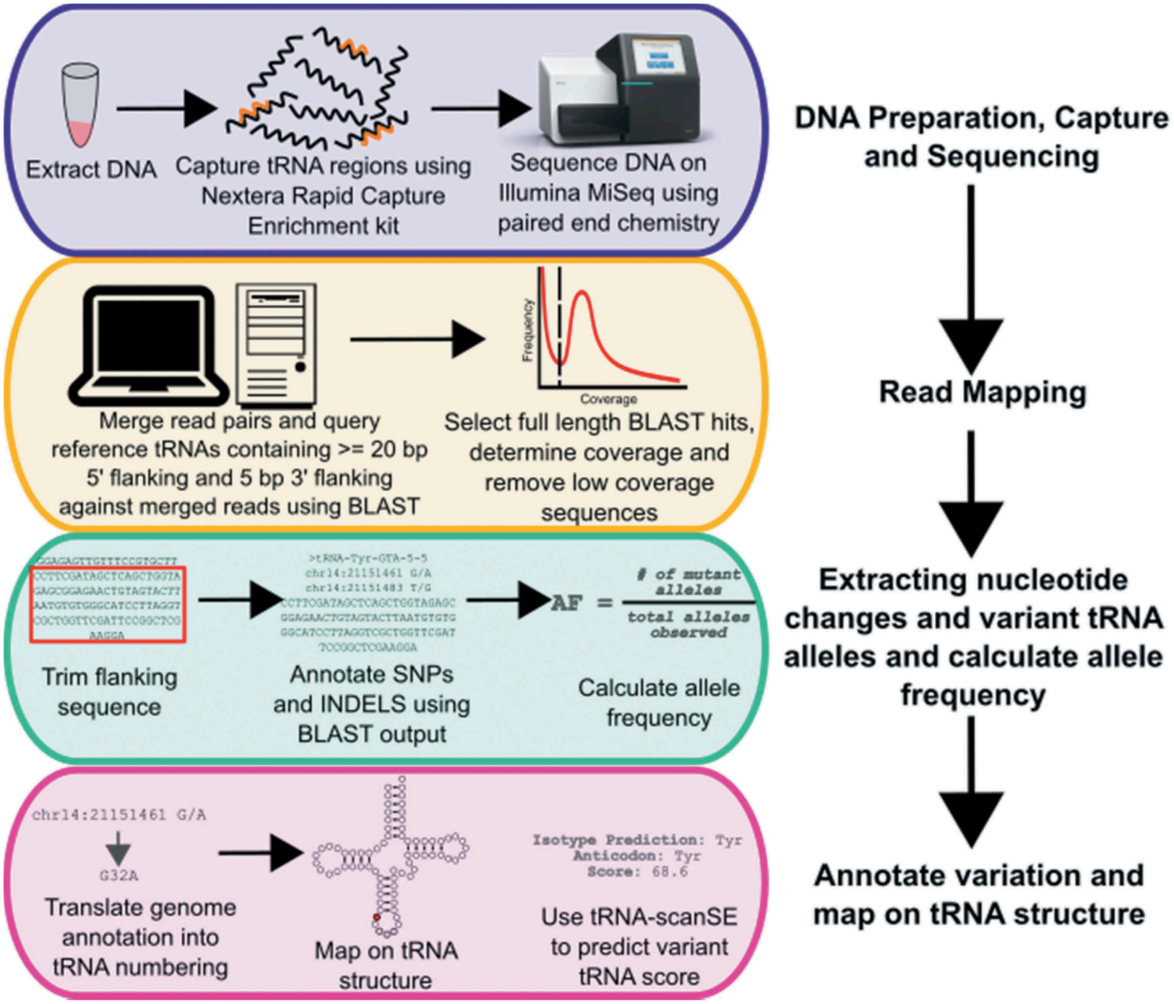
Workflow for sequencing and mapping tRNA genes, followed by variant calling, annotation and functional predictions used in this study.

Using a targeted capture panel, we aimed to identify genetic variation in tRNA-encoding genes. The workflow to capture, sequence and analyze tRNA variants is shown in Figure 2. First, we designed a capture panel specific to the 610 human tRNA genes based on tRNAscan-SE predictions [18]. The capture panel included 250 bp upstream and downstream of each tRNA gene (Table S1). We sequenced tRNA genes from 84 individuals (55 males and 29 females); sequences were derived from 48 individuals with high triglyceride levels (HTG) with no clear mutation driving the phenotype and 36 individuals with normal triglyceride levels as a way of increasing the diversity of the sequenced population.

A mean of 3.2 × 10^6^ sequence reads was obtained per individual (Figure S1A). As tRNA genes are highly similar, especially in isodecoders families, mapping reads using traditional algorithms led to mis-mapping and inaccurate variant calling. Instead, the paired-end sequences were merged (Figure S1B) and a BLAST database was created for each of the 84 individuals. To map the reads to their respective tRNA gene loci, the 610 tRNA genes from the reference genome, including 20 bp of 5ʹ flanking sequence and 5 bp of 3ʹ flanking sequence, were queried against the 84 BLAST databases. Only merged reads that covered the entire tRNA gene including flanking sequence were kept. Analyzing only full length reads also enabled identification of multiple mutations within the same allele, instead of viewing each nucleotide change independently. This is particularly important for tRNAs because of their abundant intramolecular interactions. Including flanking sequence, particularly 5ʹ sequence, in the BLAST analysis was necessary because of the identity or near identity of tRNA genes within isodecoders families. Without the 5ʹ flanking sequence, reads corresponding to 168 tRNA genes would be mis-mapped or ambiguously mapped because the gene is identical to one or more different tRNA genes. In all cases, these correspond to tRNA genes belonging to the same isodecoder family.

Based on the distribution of coverages for unique sequences (Figure S2), a 10-fold read depth cut-off was selected to exclude tRNA gene sequences that arose due to sequencing errors. With the flanking sequence included, an average of 569 tRNA genes were identified per individual from an average of approximately 45,000 full length tRNA gene reads (Figures S1C, S1D). Of these, 58 tRNA genes could not be mapped to a single locus and were annotated with all possible loci from which the read could have been derived. Over the sample set, reads mapping to 605 of the 610 tRNA genes were identified (unidentified tRNAs are listed in Table S2), with a mean sequence coverage of 66-fold (Figure S3).

### tRNA variation in the sample population

From the 84 individuals, we identified 522 unique tRNA gene sequences that differed from the reference genome, which we refer to as variants. The variants, along with the full tRNA gene sequence, can be found in supplemental file 2. The majority of tRNA variants (395 variants) occurred with a frequency of less than 5% in our sample group, which we designate as uncommon. We also found common variants: 20% of the variants occurred in our sample set with an allele frequency between 5–50%, and 4% occurred in more than half of the individuals (Figure S4). Of the total 522 variants identified, 354 localized to the high confidence tRNA genes, which are characterized by their likelihood to be expressed and participate in translation [18].

The majority (~87%) of the variants differed from the reference genome at one nucleotide; 10% of the variants had two nucleotide differences; ~2% had three nucleotide differences and less than 0.5% had four nucleotide differences. Every tRNA isoacceptor family was represented among the 522 variants. We also identified three variants in nuclear encoded mitochondrial tRNAs.

**Figure 3.**
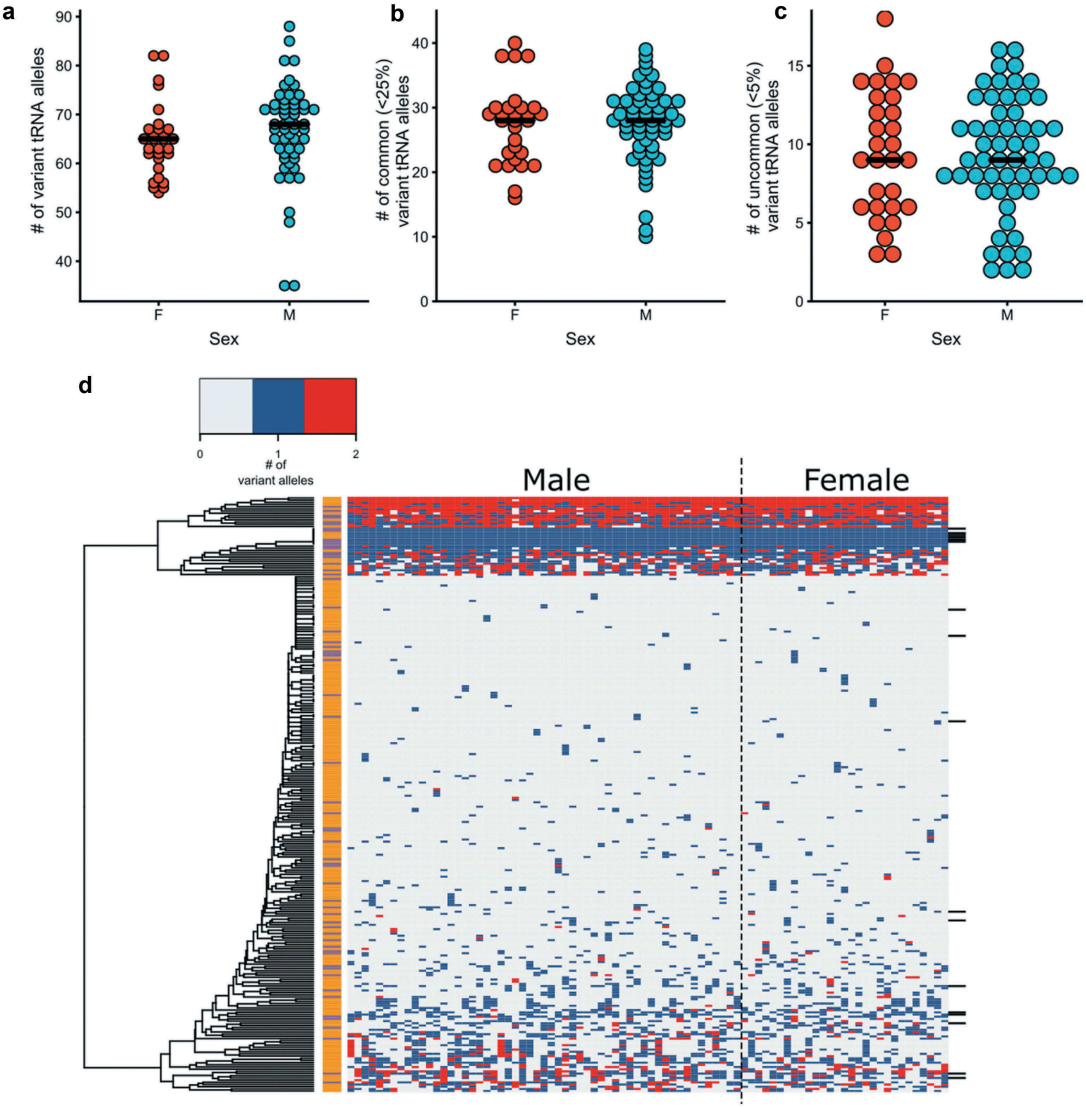
tRNA variation in individuals. Number of tRNA variants per person in males and females for (a) total variants as compared to the reference genome and (b) variants with allele frequencies less than 25% or (c) less than 5% in our sample dataset. The mean number of variants in each set is indicated (black bar). (d) Heat map of the tRNA variation profile for each individual. On the x-axis, males are grouped on the left and females on the right. Each row on the y-axis represents an individual tRNA locus or groups of tRNA where reads could not be uniquely assigned. Groups of tRNAs are denoted by black bars on the right side of the heatmap. The tRNA genes were hierarchically clustered using complete linkage and Euclidean distance. Each tRNA is labelled as either high confidence (orange) or low confidence (purple). tRNAs genes where variation was not observed are not included.

Each individual contained an average of 66 ± 9 tRNA genes that differed from the reference genome (Figure 3a). When we considered variants with allele frequencies less than 25%, each individual had 27 ± 6 tRNA variants (Figure 3b). The number of variants per individual was 9 ± 4 for the uncommon variants of less than 5% (Figure 3c and Figure S5). There was no statistical difference in total number of tRNA variants between males and females, nor was there any difference between the two subpopulations of HTG patients or controls (Figure S6).

We analyzed the variation profile for each tRNA locus across the 84 individuals that were sequenced using hierarchical clustering (Figure 3d). No clustering of high confidence versus low confidence tRNAs, nor of any isoacceptor families was observed. There was a group of highly mutated tRNA loci that clustered together. The majority of these loci have one or two predominant variants. Some also have a small number of low frequency variants. For example, tRNA-Arg-TCG-6–1, in the highly mutated tRNA set, contains five unique sequences. Two variants occur at a frequency of less than 1%, with other single variants occurring at frequencies of 10%, 25% and 30% in our sample dataset. Over 80% of the highly variant tRNAs encode low confidence tRNAs. We used K-means clustering to group individuals based upon sex or HTG levels. Both male and females and HTG and controls were intermixed, suggesting no difference in tRNA profiles between these groups (Figure S7).

**Figure 4.**
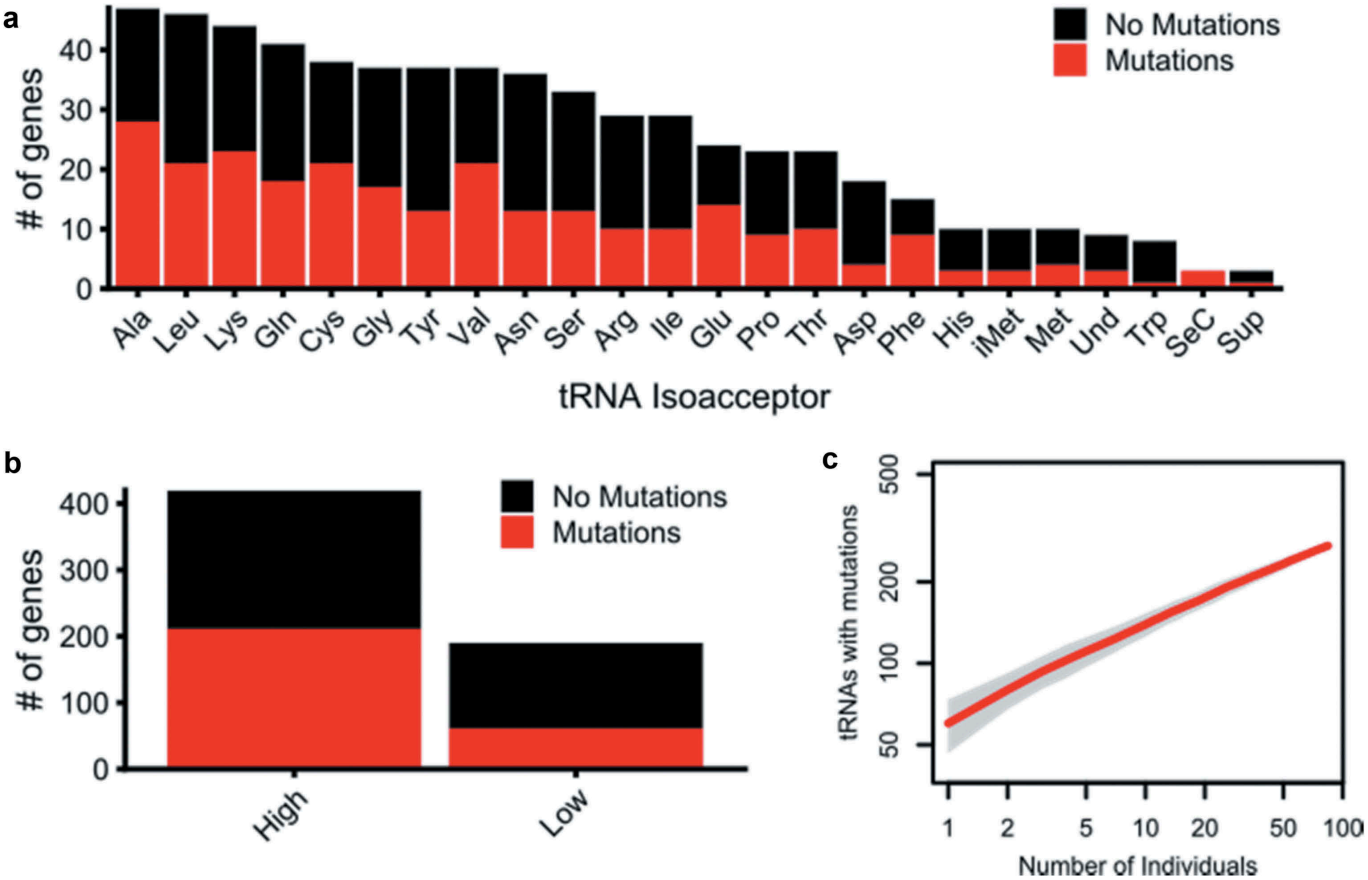
Distribution of tRNA-encoding genes with variation. Break down of tRNA genes in which we observed variation by (a) isoacceptor and (b) high confidence and low confidence annotation. In (a), ‘Und’ represents tRNAs whose identity and anticodon are undefined in the tRNA database [18]. (c) Variation accumulation curve plotted using the R package ‘vegan’ [92] with 100 subsamplings without replacement for each grouping of individuals on the x-axis. The red line represents the mean number of tRNA loci with mutations for each grouping size of individuals and the grey boundaries represent the standard deviation.

In our sample of 84 individuals, 48% (272) of the tRNA-encoding loci or groups of loci contained mutations. All isoacceptors were represented in the variant and invariant loci, with the exception of selenocysteine where we observed variants in all three genes (Figure 4a). There was no statistical difference between the proportion of variant and invariant loci for each group (*p* = 0.12). Mutations occurred at a similar frequency in high confidence and low confidence tRNAs (Figure 4b). Because of our limited sample size, we hypothesized that our analysis does not approach the full extent of tRNAs variation found in the broader population. To test this, we plotted a variation accumulation curve (Figure 4c). Based upon the continuing upward trajectory of this plot, we conclude that significantly more variation exists in the human population.

### Location of variants on the tRNA structure

**Figure 5.**
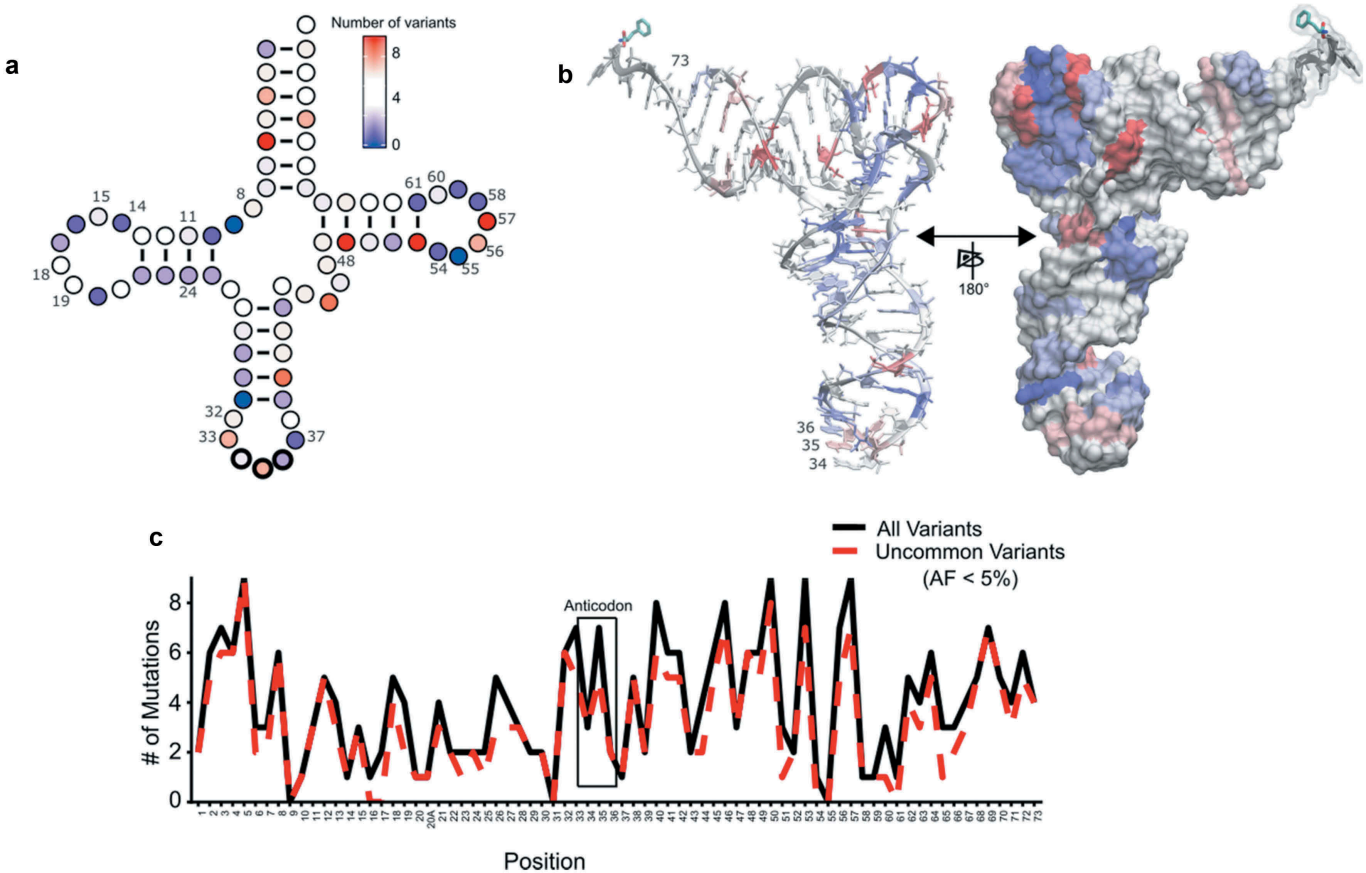
tRNA variation is distributed throughout the tRNA structure. (a) The location of all single nucleotide variants in high confidence tRNAs was mapped onto the canonical two-dimensional tRNA structure, using the canonical numbering [19]. Nucleotides colored darker blue have the least number of variants, white nucleotides have an intermediate number and red nucleotides have the most variants. Insertions, deletions and variants in the extended variable arm of serine and leucine tRNAs or within introns were not included. (b) The location of all single nucleotide variants from (a) were mapped onto the three-dimensional tRNA structure. Coloring is the same as in (a). (c) The number of variants at each position was plotted for high confidence tRNAs for alleles occurring in less than 5% of our sample population (red dotted line) and for all variant alleles identified in this study (black solid line).

In the high confidence tRNA set, we observed 291 variants with one nucleotide change. We mapped these variants onto the tRNA structure (Figure 5a and 5b). In our sample group, all positions, with the exception of positions 9, 31 and 55, had variation. On average, each position had four unique variants. We predict that the absence of mutation at nucleotides 9, 31 and 55 is due to the limited proportion of total variation that we identified rather than these being invariant positions across the larger population; variants at these sites are recorded in the 1000 Genomes Project. Thirteen variants were identified in the extended variable arms of leucine and serine tRNAs. There were two insertions, both in tRNA-Thr-TGT-6-1, six deletions in various tRNAs and nine mutations in the introns of tRNAs. Of the deletions, two occur in the acceptor stem and one occurs in the T-arm, potentially altering the conserved 7/5 base pair structure of the acceptor stem/T-arm required for interactions with the translation machinery [33]. Similar trends were observed for variants with allele frequencies less than 5% (Figure 5c).

**Figure 6.**
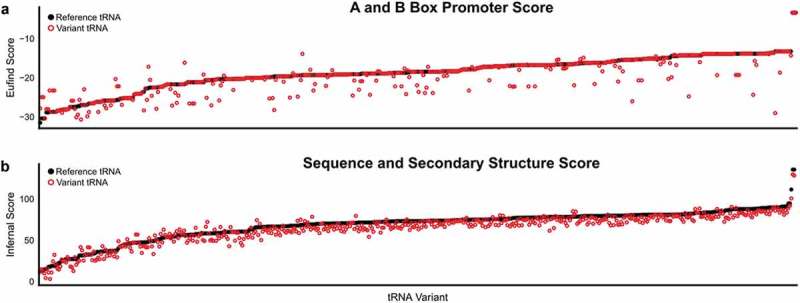
Predicted effect of tRNA variation on expression and function. The (a) EufindtRNA score and (b) Infernal score for each variant tRNA (red circle) and its reference tRNA (black circle) was computed using tRNAscan-SE [36] and rank ordered from lowest to highest reference tRNA score.

tRNA variants that result in loss of function may impact translation fidelity or protein synthesis rates by altering cellular tRNA pools [26]. Eukaryotic tRNAs are transcribed by RNA polymerase III and require internal A and B box promoters for expression. The EufindtRNA algorithm [34] scores the A and B box regions in tRNAs based off a consensus sequence. We used this score to predict the likelihood that tRNA variants would alter expression (Figure 6a). Approximately 8% and 28% of the unique variants were predicted to increase and decrease the EufindtRNA score, respectively.

Next, we used the program Infernal [35] to score the variants based upon covariance models of tRNA secondary structure and sequence consensus to predict whether the variant sequences are consistent with a proper tRNA fold and active in translation. The majority (75%) of the variations decreased the Infernal score of the tRNA, 5% were neutral, and 17% increased the score relative to the reference tRNA (Figure 6b). The Infernal tool also predicts if a tRNA gene is a pseudo tRNA. According to Lowe and Eddy [36], tRNA genes are predicted to be a pseudo tRNA if the portion of the score corresponding to the primary sequence is less than 10 bits or if the proportion of score corresponding to the secondary structure component is less than 5 bits [36]. Ten sequences have variation that changed a functional prediction to a pseudo tRNA, whereas four variants were predicted to create a functional tRNA from a putative pseudo tRNA (Table S3).

**Figure 7.**
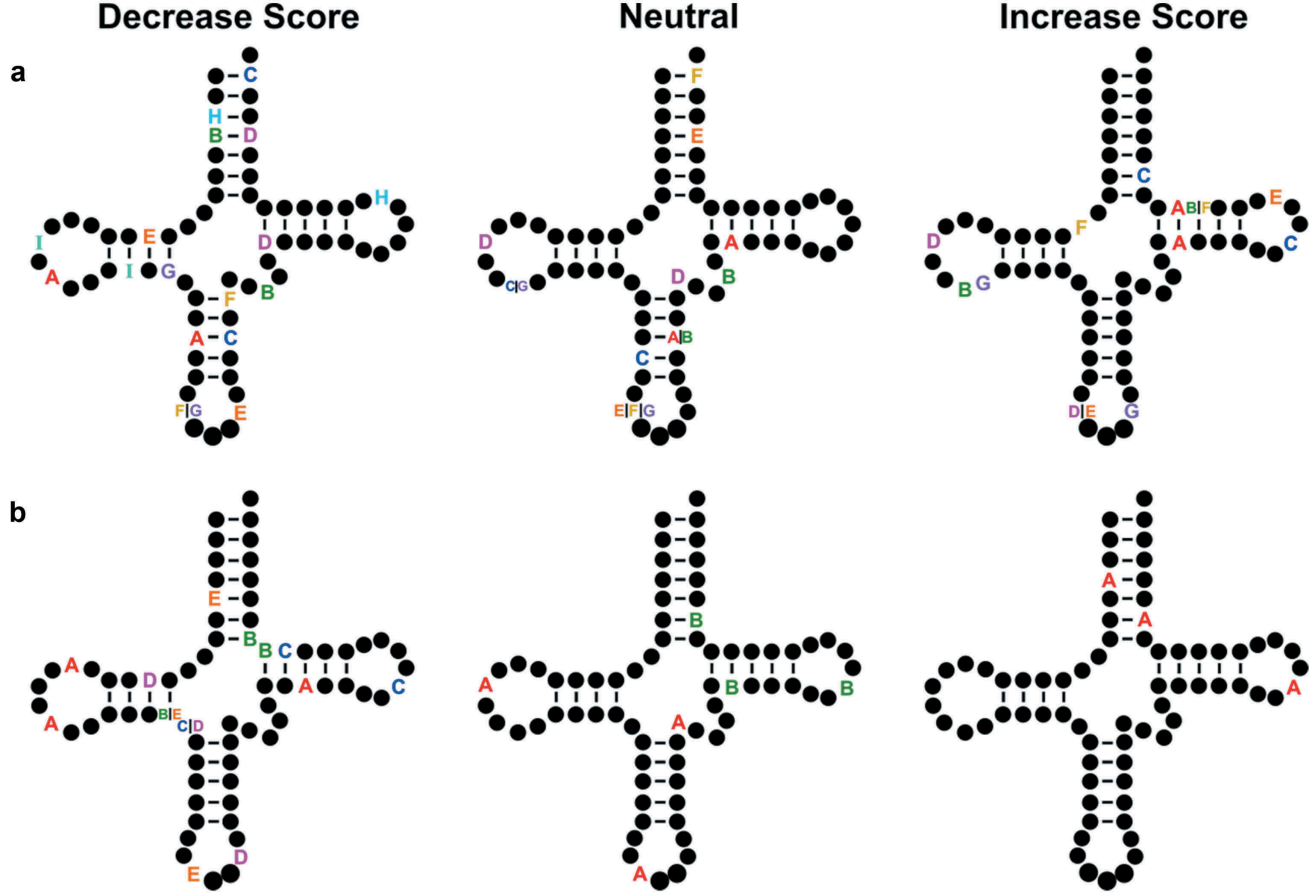
tRNA variants with multiple nucleotide changes. The Infernal score was calculated for high confidence tRNAs containing multiple nucleotide changes. Variants that decreased score are shown on the left, variants whose scores were neutral and did not change by more than 1 bit are in the middle and variants whose scores increased are on the right for variants containing (a) two or (b) three polymorphisms in the same allele. Each set of letters represent the position of single nucleotide changes within a single tRNA allele. Variants in the intron or extended variable arm are not shown.

By aligning paired-end reads we were able to identify tRNAs with multiple nucleotide changes compared to the reference genome. There were 27 high confidence tRNA alleles with two nucleotide changes and eight containing three nucleotide changes. The majority of the double mutants occur as pairs in the anticodon arm and D-arm. Eight contain at least one nucleotide change in the T-arm. Seven of the variants with two nucleotide changes had an increased Infernal score compared to the reference tRNA while 13 variants had a decreased score. Seven variants did not alter the score by more than 1 bit. The majority of the variants with three nucleotide changes had a decreased score. Two variants with three nucleotide changes were neutral and one variant increased the score. Figure 7 shows the variants mapped on the two-dimensional tRNA structure.

In addition to loss-of-function mutations, tRNA variation can cause gain of function through mistranslation of the genetic code. Unlike other isoacceptors, the anticodon of alanine, serine, leucine and selenocysteine tRNAs has no role in aminoacylation. If mutated and expressed, these tRNA variants could result in mistranslation [37]. We looked for variants with the potential to mistranslate either through mutations to the anticodon or by possessing a G3:U70 base-pair, the major identity element for AlaRS [37–40]. Six variants with mutations at position 3 create a G3:U70 base-pair in tRNA^Gly^ (Table 1). Three of the variants had single mutations. The G3:U70 variants in the high confidence tRNA-Gly-GCC-1-5 and tRNA-Gly-CCC-1-1 occur at a frequency of ~1 and 5%, respectively. Another variant (in tRNA-Gly-CCC-5-1) altered a C3:U70 mismatch to G3:U70 increasing the Infernal score of the tRNA.

**Table 1.** Potential mistranslating tRNAs with G3:U70 identity elements.

tRNA	Variant	Allele frequency	Position	High confidence tRNA^a^
tRNA-Gly-CCC-1-1	chr1:16872502 T/C	0.052	3	Yes
tRNA-Gly-CCC-5-1	chr1:17053782 C/G	0.215	3	No
tRNA-Gly-GCC-1-5	chr21:18827175 T/C	0.012	3	Yes
tRNA-Gly-CCC-1-1	chr1:16872502 T/C chr1:16872448 A/G	0.046	3, 59	Yes
tRNA-Gly-CCC-5-1	chr1:17053782 C/G chr1:17053826 C/T	0.058	3, 49	No
tRNA-Gly-CCC-5-1	chr1:17053782 C/G chr1:17053835 -/C	0.012	3, 58	No

^a^As noted in GtRNAdb [18].

We identified 18 variants with changes in the anticodon (Table 2), 14 being in the high confidence set. Of the single nucleotide variants, four of the anticodon variations changed G to A at position 34. These do not alter the amino acid accepting identity of the tRNA, although they may expand the decoding potential of the tRNA if A34 is modified to inosine [41,42]. Mutations at position 35 and 36 change tRNA decoding identity. Two notable variants of this type are in tRNA^Ala^ and tRNA^Ser^. The G to C change at position 35 of tRNA^Ala^ potentially results in decoding of glycine codons with alanine, whereas, G35A in tRNA^Ser^ would decode phenylalanine codons with serine.

**Table 2.** tRNA variants with anticodon substitutions.

tRNA	Variant	Allele frequency	Position	WT anticodon	WT codon identity	Variant anticodon	Variant codon identity	High confidence tRNA^a^
**Synonymous variants**								
tRNA-Asn-GTT-2-3	chr10:22518477 C/T	0.006	34	GTT	Asn	ATT	Asn	Yes
tRNA-Asn-GTT-2-6	chr19:1383596 G/A	0.012	34	GTT	Asn	ATT	Asn	Yes
tRNA-Asn-GTT-15-1	chr1:149284542 C/T	0.006	34	GTT	Asn	ATT	Asn	No
tRNA-Gly-GCC-2-2	chr2:157257697 C/T	0.006	34	GCC	Gly	ACC	Gly	Yes
tRNA-Lys-TTT-7-1	chr6:28715537 T/Cchr6:28715554 T/Cchr6:28715564 C/G	0.006	17, 34, 44	TTT	Lys	CTT	Lys	Yes
tRNA-Arg-CCT-5-1	chr16:3243922:C/Achr16:3243942:T/C chr16:3243951:C/T	0.307	5, 25, 34	CCT	Arg	TCT	Arg	Yes
**Non-synonymous variants**								
tRNA-Thr-AGT-5-1	chr17:8042808 C/T	0.006	35	AGT	Thr	AAT	Ile	Yes
tRNA-Ser-AGA-2-3	chr6:27463627 G/A	0.030	35	AGA	Ser	AAA	Phe	Yes
tRNA-Asp-GTC-3-1	chr6:27551273 A/T	0.006	35	GTC	Asp	GAC	Val	Yes
tRNA-Val-AAC-6-1	chr6:28703244 T/C	0.536	35	AAC	Val	AGC	Ala	Yes
tRNA-Ala-AGC-6-1	chr6:28779887 C/G	0.085	35	AGC	Ala	ACC	Gly	Yes
tRNA-Cys-GCA-10-1	chr7:149074639 G/A	0.006	35	GCA	Cys	GTA	Tyr	Yes
tRNA-Arg-TCG-6-1	chr9:112960837 C/T	0.006	35	TCG	Arg	TTG	Gln	Yes
tRNA-Gly-TCC-2-6	chr1:161500938 C/A	0.006	36	TCC	Gly	TCA	STOP	Yes
tRNA-Gly-TCC-2-6	chr1:161500938 C/G	0.006	36	TCC	Gly	TCG	Arg	Yes
tRNA-Ala-AGC-18-1tRNA-Ala-AGC-18-2	chr6:26728291 C/T	0.003	36	AGC	Ala	AGT	Thr	No
tRNA-Cys-GCA-chr8-6	chr8:111946808 A/G	0.015	36	GCA	Cys	GCG	Arg	No
tRNA-Ser-ACT-1-1	chr6:27261700 C/Gchr6:27261706 C/T	0.024	29, 35	ACT	Ser	ATT	Asn	No

^a^As noted in GtRNAdb [18].

## Discussion

tRNA variants represent an untapped source of genetic diversity, yet the extent of tRNA variation has been a difficult problem to resolve. In part this is due to the complexity associated with sequencing heavily modified tRNAs using cDNA-like protocols. Furthermore, the large number of closely related tRNA-encoding genes creates alignment and mapping challenges when using traditional read mapping programs, problems which are exacerbated by short-read technology sequencing platforms. These are critical issues because of the differential expression of isodecoders in cells and tissues [31] and the fact that multiple mutations within the same allele can impact tRNA function differently as compared to viewing each mutation in isolation.

Analysis of the 1000 Genomes Project has led to the prediction of 1 to 2 variant tRNAs per person compared to the reference genome [32]. Our analysis has demonstrated that the average individual has ~27 tRNA-encoding alleles that differ from the reference genome, which occur at an allele frequency less than 25%, and nine, which occur at a frequency less than 5%.

By deep sequencing tRNA genes captured from individuals, we demonstrate that each individual has a relatively unique tRNA variation profile. Three key aspects of our approach allowed the identification of increased variation. First, the custom capture panel enriched for sequences containing tRNA-encoding genes and resulted in, on average, greater than 60x coverage. The 1000 Genomes Project aimed to sequence each sample at 4x coverage, which only allows detection of variants with frequencies greater than 1% in their sample group [43]. In our sample group, 254 out of our 522 unique variants were found only once and therefore would have been excluded. Secondly, upon analyzing tRNA gene sequences from the 1000 Genomes Project, Parisien et al. [32] recognized that the similarity of tRNA isodecoders makes their unique mapping difficult. They found regions of tRNA isodecoders with more sequence coverage compared to other regions and correlated the coverage increase to a degeneracy index based upon the similarity of each region to isodecoders of the same tRNA. The 1000 Genomes Project data thus only allowed identification of new tRNA isodecoders, not the unique tRNA gene loci from which they are derived. By merging paired end reads and selecting reads that contain the full length tRNA plus gene specific flanking sequence, we were able to uniquely map reads to 94% of the tRNA genes and thus we were able to annotate a greater number of variants. Only 58 tRNAs, representing 23 groups of highly similar tRNAs, could not be uniquely mapped. It is possible to map these reads by extending the flanking sequence, but the increased required read length correspondingly reduces the coverage below a reliable threshold.

The ability to merge paired-end reads into sequences that spanned entire tRNA sequences allowed us to identify multiple polymorphisms within the same allele. To our knowledge, this is the first systematic analysis of human tRNA alleles with multiple mutations. More than one nucleotide change was found in 69 of the unique variants we identified. The recognition of alleles with multiple polymorphisms is particularly important for tRNAs because approximately 50% of the nucleotides are involved in base-pair interactions, with other nucleotides interacting to facilitate the tertiary fold of the tRNA. In addition, many identity elements that direct tRNA aminoacylation consist of base-pairs or structural motifs in the tRNA. Multiple variations may simply further decrease function as compared to a single variation. However, if two nucleotide changes are positioned so as to restore a base-pair or tertiary interaction, the secondary SNP could restore function or in the case of, for example the G3:U70 base-pair in tRNA^Gly^ noted above, lead to altered function. In this regard, when analyzing tRNA variants it is crucial that sequences be analyzed as intact alleles.

While we have identified more tRNA variation than previously seen, our limited sample set suggests that there is much more human tRNA variation to be identified in the larger human population. Also suggesting more variation is the fact that our sample population is limited to individuals living within London, Ontario and the surrounding region. We anticipate future sequencing studies, including additional ethnic groups, will reveal even more tRNA variation.

Many of the 522 unique variants we identified likely result in loss of function as suggested by their decreased Infernal score. These variants have the potential to alter tRNA pools, leading to translation errors and proteome disruption. The impact of altered tRNA pools is demonstrated in the effects of silent mutations on protein expression and folding [44–46]. In one example, a silent mutation in *CFTR*, the gene encoding the chloride ion channel that is linked to cystic fibrosis, results in decreased protein levels specifically in bronchial epithelial cells [45]. Kirchner et al. [45] found that the silent mutation created a codon that corresponded to a low abundance tRNA in the lung. Ribosome stalling occurs during the search for the low abundance tRNA, resulting in mis-folded protein that is degraded. Correspondingly tRNA variation may deplete a tRNA isodecoder pool causing ribosome stalling and alter the proteome.

tRNA pools also regulate translational speed to distribute ribosome traffic on mRNA and to assist protein folding [47]. For highly expressed genes, the 5ʹ most codons often correspond to tRNAs that are low abundance [48–50]. Tuller et al. [49] predicted and Ingolia et al. [51] demonstrated that translation is slow in this region, allowing uniform ribosome spacing which would prevent collisions and allow for efficient protein synthesis. In addition, codons corresponding to rare tRNAs often cluster within genes to create slowly translated sections that give time for the already translated peptide to fold [46,52,53]. In yeast, altering tRNA pools by deleting tRNA genes upregulates the translation machinery and decreases cellular fitness [54].

Mistranslation is a general feature of the translation process which has a basal error rate of approximately one mis-incorporated amino acid in every 10^4^ to 10^5^ codons [55,56]. Many tRNA variants dramatically increase mistranslation in bacterial, fungal, and mammalian cell culture systems [37,39,57–60]. For example, we identified a variant of yeast tRNA^Pro^ that results in ~5% proteome wide alanine for proline mistranslation with a minimal effect on cell growth in yeast [39]. The same tRNA results in ~3% mistranslation in mammalian cells with virtually no effect on cell viability [37]. We therefore scanned the human tRNA sequences for variants with the potential to mistranslate the genetic code. We found 6 unique variants that created a G3:U70 element in the acceptor stem of glycine tRNAs. This is the major identity element for recognition by the alanine tRNA-aminoacyl synthetase with the G3:U70 base-pair found exclusively in tRNA^Ala^. Transplantation of the G3:U70 element onto non-alanine tRNAs leads to aminoacylation with alanine [38,40,57]. We thus predict that the tRNA^Gly^ G3:U70 variants will mistranslate alanine at glycine codons. The G3:U70 example also demonstrates that while Infernal score is a good indicator of the likelihood a tRNA will fold into the proper structure, mutation to identity elements that might alter decoding must be considered separately.

In addition, we identified variants with mutations to their anticodon which could potentially lead to altered decoding. For many isoacceptors the anticodon is the major identity element for aminoacylation and mutation of the anticodon leads to a corresponding change in aminoacylation. However, the anticodon is not an identity element for alanine, leucine, serine and selenocysteine tRNAs. In model systems, changes to the anticodons of these tRNAs result in mistranslation [59,60]. Four specific examples we identified are anticodon mutations in serine tRNAs that change the decoding of serine codons to phenylalanine or asparagine and two anticodon mutations in alanine tRNAs that change the decoding of alanine codons to glycine or threonine. Though tolerated, mistranslation disrupts the proteome and leads to proteotoxic stress [39,60–64]. Furthermore, in human cell culture and in chick embryos, engineered serine tRNAs with non-serine anticodons decrease cell division, induce apoptosis and activate the unfolded protein response [58].

Whether through loss of function or mistranslation, tRNA variants have the potential to disrupt the proteome. The loss of proteostasis is a hallmark of many diseases, including neurodegenerative diseases and cardiomyopathies [65,66]. We hypothesize that some of the many tRNA variants in the population could be modulators of disease through their ability to further disrupt the proteome and reduce tolerance to a primary mutation. One example of this is the synergistic effect of a point mutation in tRNA^Arg^, which enhances neurodegeneration caused by a mutant *GTPBP2* gene, a ribosome recycling factor, in mice [27]. The same tRNA^Arg^ mutation is also found in the human population [26]. The hypothesis is further supported by the association of tRNA modifying enzymes and error prone tRNA synthetases with disease. For example, mutations in tRNA modifying enzymes lead to type 2 diabetes [67,68], microcephaly and neurological disorders [23] and defects in haematopoiesis [69] whereas mutations affecting aminoacyl-tRNA synthetase editing cause neurodegeneration [64] and cardiac fibrosis and dysfunction in mice [70].

The importance of identifying locus specific sequence differences is emphasized by the differential expression of tRNA genes. Danielson et al. [71] and Umu et al. [72] identified 356 and 411 unique tRNAs, respectively, in plasma using RNA sequencing approaches. It is estimated that cells express between 300 and 400 tRNA genes, based on microarray [73–76] or RNA sequencing approaches [31,77–79]. This number is consistent with RNA polymerase III and TFIIIC occupancy [80–82]. Levels and patterns of tRNA isoacceptor and isodecoder expression are tissue specific [73] with cancerous and proliferating cells showing overall tRNA expression increases as much as 10-fold [74–76,83]. This differential expression of tRNA genes in human cells agrees with dynamic expression in model organisms. In *Caenorhabditis elegans* tissue and temporal differences in expression are seen for otherwise identical tRNA genes, depending on the genomic context in which they are found [84]. In yeast, stress alters tRNA expression [85,86] and similar stress responsive changes in tRNA pools are seen in human cells as the result of retrograde transport between cytosol and nucleus of specific tRNAs [87].

Detailed analysis of the tRNA genome and properly powered association studies will be required to develop links between tRNA variants and disease. Our analysis and scripts allow comparisons of the number of variants per isoacceptor and/or isodecoder. Furthermore, they allow comparison of the number of variants that increase or decrease tRNA score. This added complexity is required because proteome disruption might not be linked to a specific tRNA or isodecoder but rather be associated with the sum of tRNA variation that disrupt proteostasis. Overall, the increased amount of tRNA variation identified in this study demonstrates that tRNA genes are an untapped source of genetic diversity with the potential to have profound effects on the proteome and on human health and disease.

## Materials and methods

### Design of tRNAseq panel

The Nextera Custom Enrichment kit (Illumina, San Diego, CA) was used to capture genomic regions corresponding to 610 tRNA-encoding genes [coordinates obtained April 2017 from GtRNAdb 2.0; [18], Table S1]. A 250 base-pair pad was included surrounding each captured region. Chromosome scaffold coordinates were obtained from the University of California Santa Cruz genome browser using the February 2009 GRCh37/hg19 genome build [88] and were submitted to the Illumina Online Design Studio (Illumina, San Diego, CA). A total of 8081 target-specific probes were designed with an average length of 518 bps.

### Sample selection, DNA isolation and sequencing parameters

Eighty-four individuals were sequenced in this study. Forty-eight individuals with hypertriglyceridemia (HTG) had plasma triglyceride levels greater than 10 mmol/L with no genetic explanation. Specifically, the subjects do not have CNVs or deleterious variants in triglyceride-associated genes: *LPL, APOC2, APOA5, LMF1, GPIHBP1, GPD1* and *GALNT2*. The other 36 individuals were controls with triglyceride levels less than 2 mmol/L. The project was approved by the human ethics review board of Western University (#07920E). DNA was isolated at the Blackburn Cardiovascular Genetics Laboratory from 4 mL of blood using the Puregene® DNA Blood Kit (Gentra Systems, Qiagen Inc., Mississauga, ON, Canada). DNA quantities and quality were measured using a NanoDrop-1000 Spectrophotometer (Thermo Fisher Scientific, Waltham, MA, USA) and samples diluted to 30 ng/μL. Libraries containing 12 samples each were prepared at the London Regional Genomics Centre as described in the Nextera Rapid Capture Enrichment protocol and then sequenced on an Illumina MiSeq (Illumina, San Diego, CA, USA). The data was output as FASTQ files containing the sequencing reads with corresponding quality score determined by the sequencer for each nucleotide. The raw sequencing reads were deposited as FASTQ files and can be found at the European Nucleotide Archive under accession number PRJEB32805.

### Mapping, calling and annotating tRNA variants

To map sequencing reads to their corresponding tRNA gene, paired end reads were merged using Usearch [v11.0.667_i86linux32; [89]] and BLAST databases were constructed for each of the samples using the BLAST suite from NIH (v2.2.31). The reference set of tRNAs annotated in GtRNAdb [18] downloaded February 2019 from the UCSC genome browser (GRCh37/hg19) based on the coordinates used to design the tRNA capture panel and containing an additional 20 bp of 5ʹ flanking sequence and 5 bp of 3ʹ flanking sequence were queried against each BLAST database (locus-specific query sequences can be found in Supplemental File 1) using a 95% identity threshold. A custom Perl script was used to parse through the BLAST output and select only the reads that contained a full length hit (including flanking sequence and the tRNA gene). Reads were annotated with their genomic locus corresponding to the February 2009 GRCh37/hg19 genome build. If a read was a match for two or more loci and could not be further distinguished, it was annotated with all possible loci. Next, the flanking sequence was trimmed, leaving only the tRNA gene. Variants from the reference genome were determined from the ‘btop’ output of the BLAST. All variants from the 84 individuals sequenced were collected and allele frequencies calculated by determining the total number of alleles observed at a locus (or group of loci for tRNA genes that could not be unambiguously mapped) and dividing by the frequency the variant allele was observed in the sample group. We assumed that if only one unique allele was observed at a locus, the individual was homozygous at that locus. Finally, we developed a custom Perl script to annotate the variants onto the canonical tRNA structure based on the genomic coordinates of the variants, the structure of the tRNA annotated in the GtRNAdb and the canonical tRNA numbering as described by Sprinzl et al. [19]. Eufind and Infernal tRNA scores were calculated for each variant using tRNAscan-SE version 2.0 [36]. The Perl scripts used in this study to map and analyze variants can be found on Github [90].

### Statistical analysis

Statistical comparisons of the number of mutations per individual for males and females and HTG and control individuals were performed using the unpaired Welch’s t-test function in R. Comparisons of the number of genes mutated for all isoacceptors and between high and low confidence tRNAs were assessed using the proportion test function in R. Clustering of the amount of variation per tRNA gene (or group of tRNA genes) was done using the ‘Heatmap.2ʹ function in the gplots package [91] in R. The variation accumulation plot was generated using the R package ‘vegan’ [92]. Significance for any statistical test was assessed at an α = 0.05 after Bonferroni correction. The R code used to test significance and create plots can be found on Github [90].
