# Supplementary material for: Targeted sequencing reveals expanded genetic diversity of human transfer RNAs
Source: RNA Biol. 2019 Aug 13;16(11):1574–85. doi: 10.1080/15476286.2019.1646079 (PMC6779403; doi:10.1080/15476286.2019.1646079)
Supplement: Supplemental Material [file krnb-16-11-1646079-s002.zip › KRNB_A_1646079/downloadFromZipFile.pdf]

SUPPLEMENTAL FIGURES

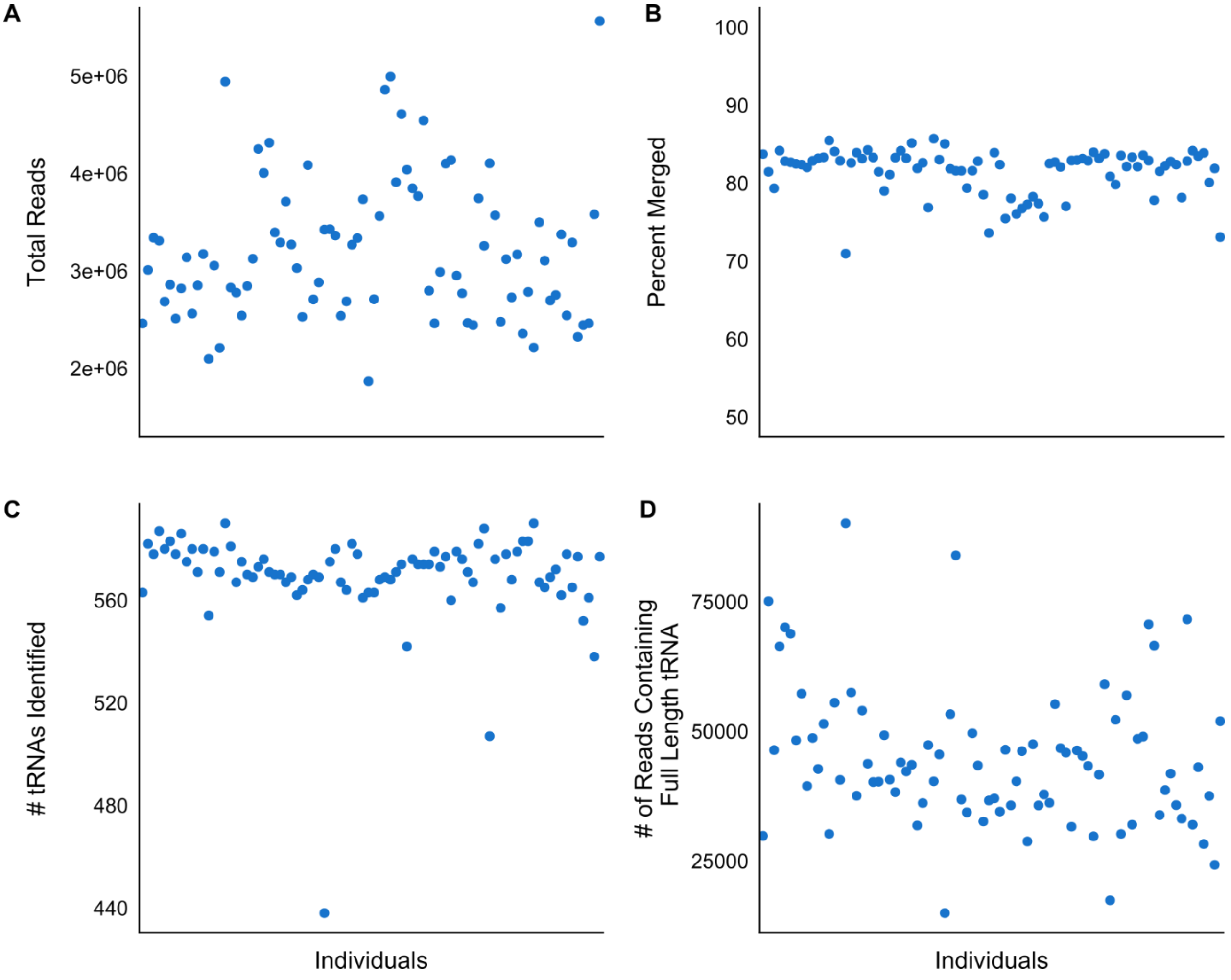

Figure S1. Summary of the analysis of sequencing reads for the tRNA sequencing capture panel. (A) Total reads per individual sequenced (B) Percent of reads successfully merged using Usearch for each sample. (C) Total number of tRNAs with greater than 10x coverage identified from the 610 possible tRNA genes for each sample. (D) Number of merged reads containing 20 bp 5' flanking sequence, 5 bp 3' flanking sequence and a full length tRNA gene.

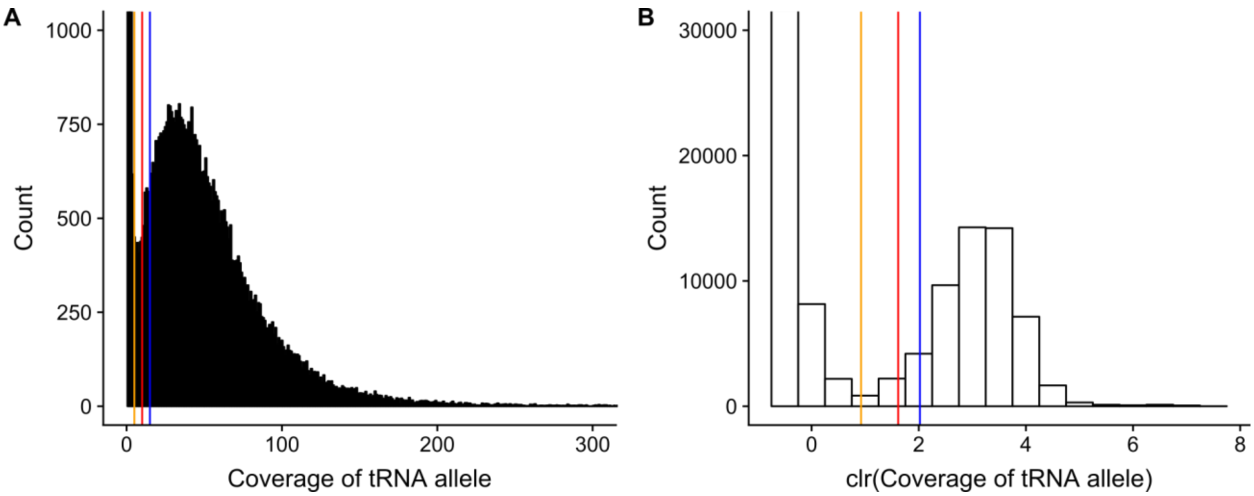

Figure S2. Distribution of coverages for unique tRNA gene sequences. Raw coverages (A) and the center log ratio transformed coverages (B) for all unique sequences observed from 84 sequenced samples. Blue line represents a coverage of 15x, red line represents a coverage of 10x and yellow line represents a coverage of 5x.

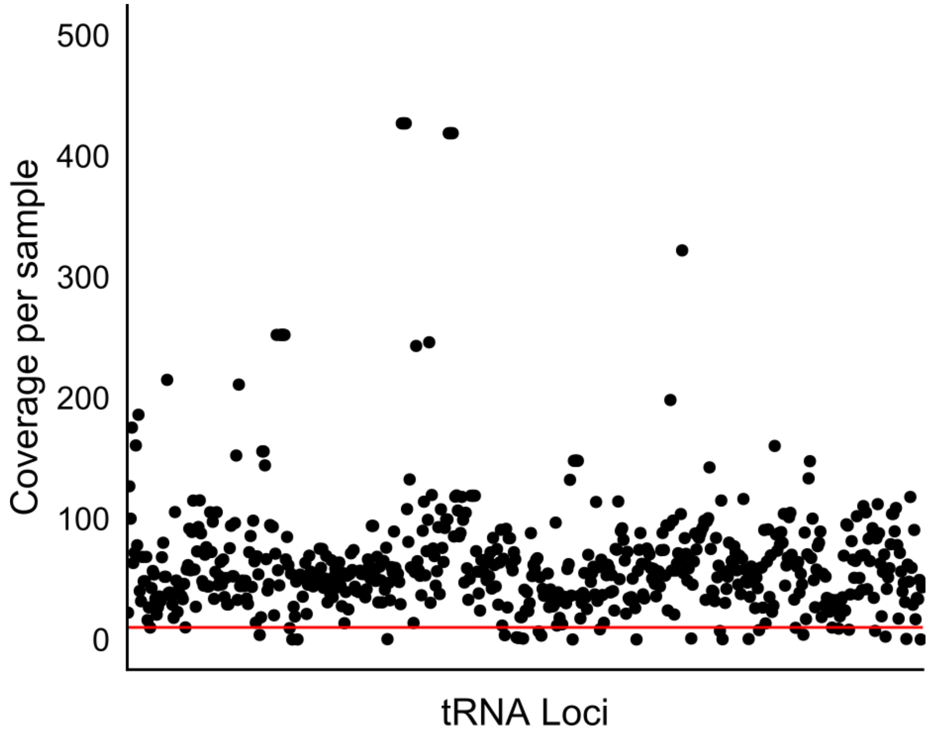

Figure S3. Average coverage per individual for all 610 tRNA loci. One loci had a coverage value over 500x and is not shown on this graph. Red line represents 10x coverage, the cutoff used in this study.

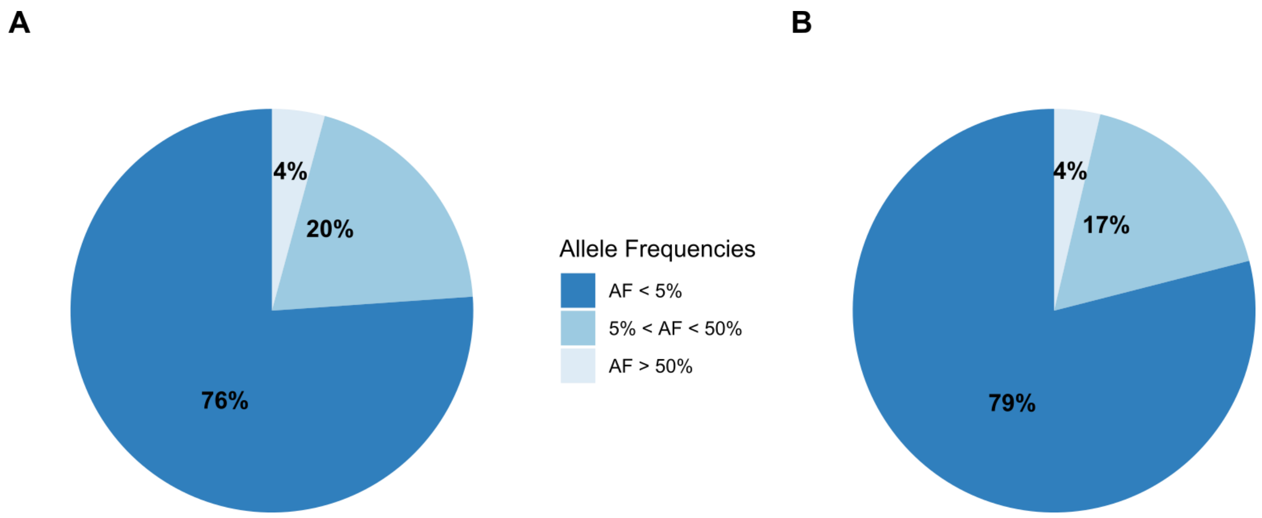

Figure S4. Distribution of allele frequencies for (A) all 522 unique tRNA variants and (B) the 354 tRNA variants that occur in high confidence tRNAs.

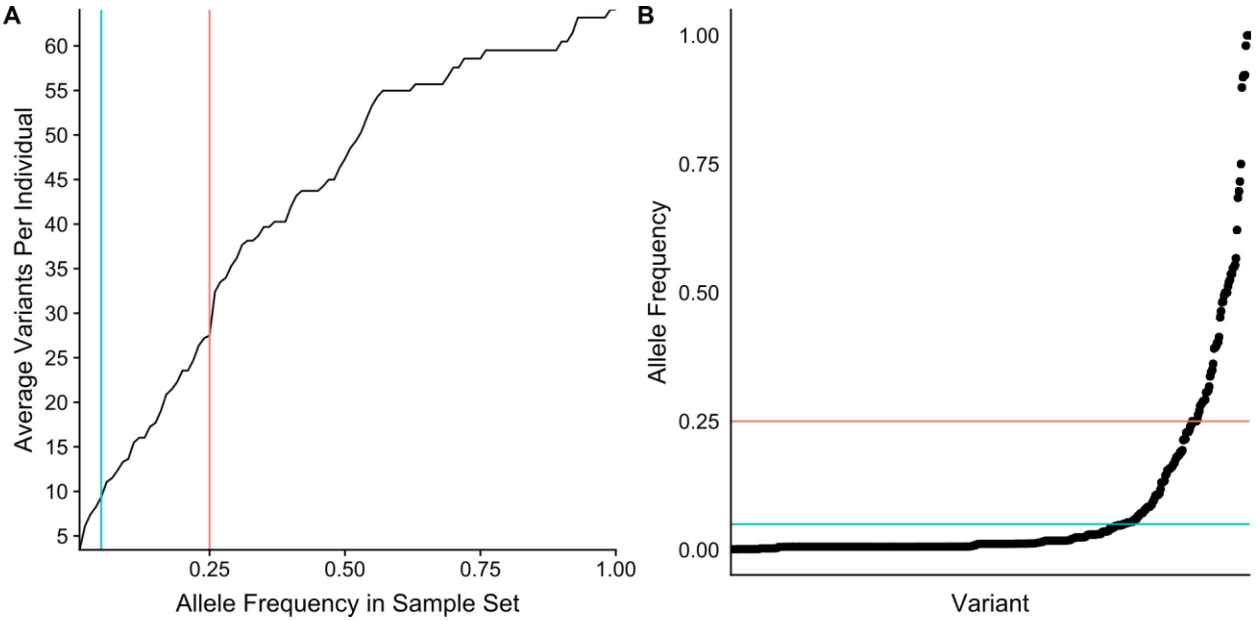

Figure S5. (A) Average number of tRNA variants per individual at different allele frequency cut-offs. The blue line denotes an allele frequency of 5% and the red denotes an allele frequency of 25%. We designate any variant occurring < 5% in our sample set to be uncommon. (B) Allele frequency for all 522 tRNA variants. The blue line denotes an allele frequency of 5% and the red denotes an allele frequency of 25% as in (A).

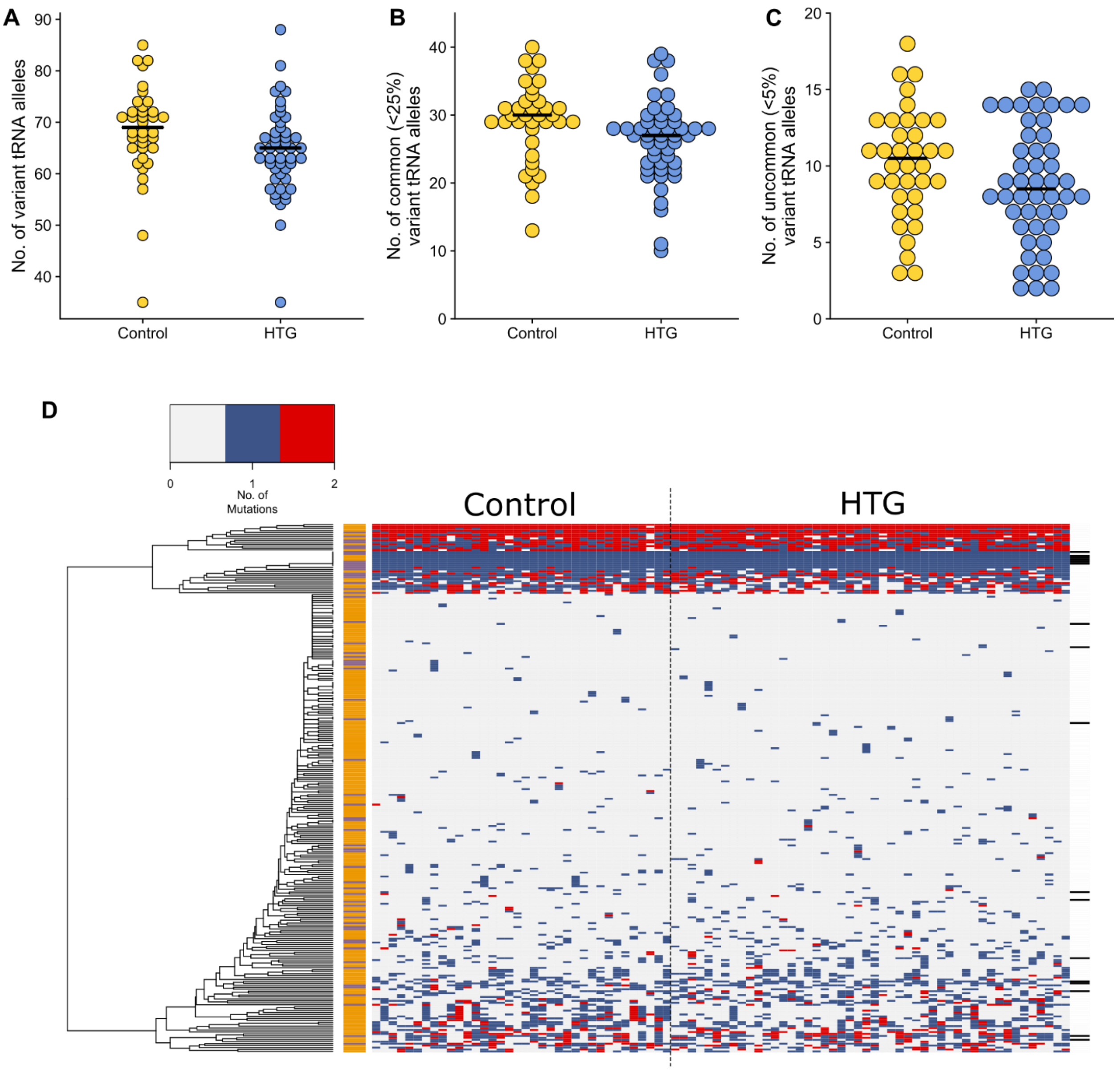

Figure S6. tRNA variation in individuals. Number of tRNA variants per person in high triglyceride (HTG) and control groups for (A) total variants as compared to the reference genome and (B) variants with allele frequencies less than 25% or (C) less than 5% in our sample dataset. The mean number of variants in each set is indicated (black bar). (D) Heat map of the tRNA variation profile for each individual. On the x-axis, control individuals are grouped on the left and HTG individuals on the right. Each row on the y-axis represents an individual tRNA locus or groups of tRNA where reads could not be uniquely assigned. Groups of tRNAs are denoted by black bars on the right side of the heatmap. The tRNA genes were hierarchically clustered using complete linkage and Euclidean distance. Each tRNA is labelled as either high confidence (orange) or low confidence (purple). tRNAs genes where variation was not observed are not included.

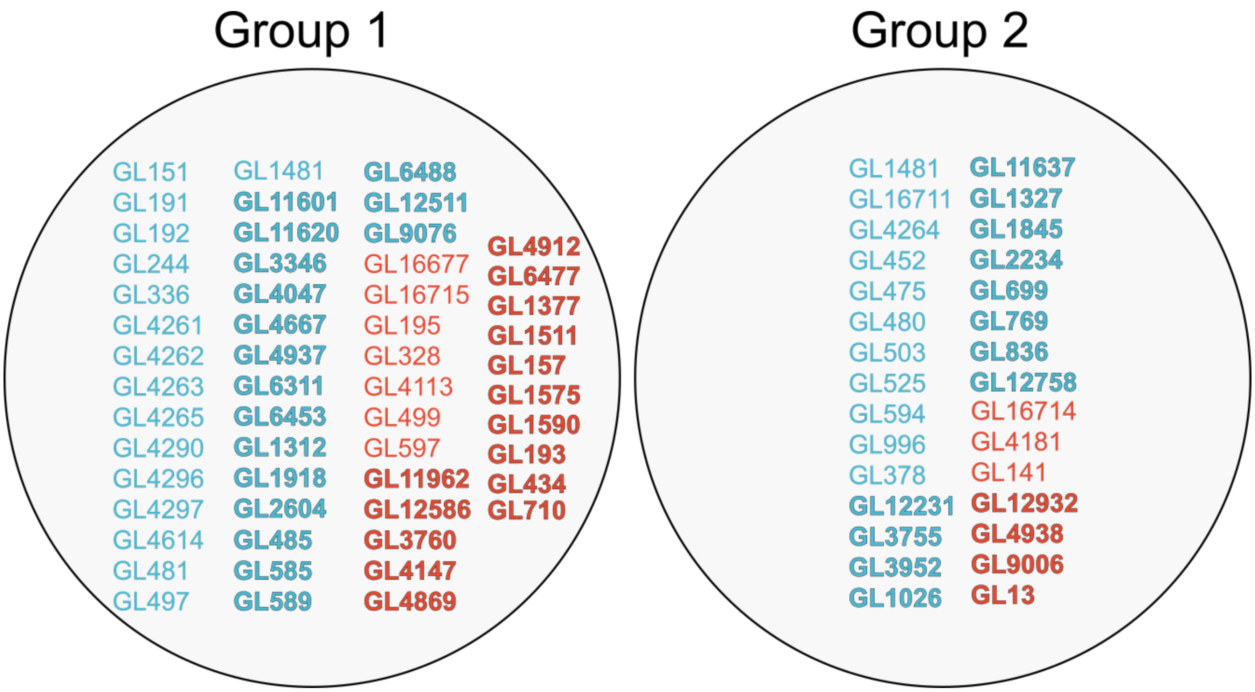

Figure S7. K-means clustering of the tRNA profiles of individuals sequenced in this study. Individuals were clustered into two groups. Males are colored blue; females are colored red. Bolded individuals are in the HTG sample group.

SUPPLEMENTAL TABLES

Table S1. Genomic loci targeted using the custom capture panel.

Chr	Start[a]	Stop[a]	tRNAscan-SE ID	Size (bp)
1	566126	566691	chr1.trna127	565
1	4626898	4627462	chr1.trna1	564
1	7990089	7990658	chr1.trna126	569
1	16846830	16847403	chr1.trna125	573
1	16858643	16859216	chr1.trna124	573
1	16861524	16862095	chr1.trna123	571
1	16872184	16872754	chr1.trna122	570
1	16873910	16874482	chr1.trna121	572
1	17004516	17005086	chr1.trna120	570
1	17006251	17006823	chr1.trna119	572
1	17053530	17054100	chr1.trna2	570
1	17186443	17187015	chr1.trna3	572
1	17188166	17188736	chr1.trna4	570
1	17198828	17199399	chr1.trna5	571
1	17201708	17202281	chr1.trna6	573
1	17215922	17216495	chr1.trna7	573
1	36325428	36326086	chr1.trna118	658
1	39969946	39970515	chr1.trna8	569
1	55423292	55423864	chr1.trna117	572
1	55838861	55839429	chr1.trna9	568
1	93981584	93982156	chr1.trna116	572
1	94312879	94313463	chr1.trna10	584
1	94399260	94399823	chr1.trna11	563
1	143663308	143663881	chr1.trna115	573
1	143689778	143690351	chr1.trna12	573
1	143879582	143880155	chr1.trna114	573
1	144301361	144301934	chr1.trna13	573
1	144308364	144308937	chr1.trna113	573
1	144481590	144482163	chr1.trna14	573
1	144488593	144489166	chr1.trna112	573
1	144839186	144839757	chr1.trna111	571
1	145379070	145379641	chr1.trna110	571
1	145385479	145386054	chr1.trna15	575
1	145395272	145395844	chr1.trna109	572
1	145396631	145397202	chr1.trna108	571
1	145397614	145398185	chr1.trna107	571
1	145398983	145399554	chr1.trna106	571
1	145950746	145951316	chr1.trna16	570
1	145963054	145963625	chr1.trna17	571

1	145978784	145979357	chr1.trna105	573
1	146476510	146477081	chr1.trna104	571
1	146544523	146545094	chr1.trna18	571
1	147492464	147493034	chr1.trna19	570
1	147504788	147505359	chr1.trna20	571
1	147520517	147521090	chr1.trna103	573
1	147716488	147717059	chr1.trna102	571
1	147718779	147719352	chr1.trna21	573
1	147737132	147737703	chr1.trna101	571
1	147753221	147753792	chr1.trna22	571
1	147774595	147775166	chr1.trna100	571
1	147780780	147781351	chr1.trna99	571
1	147800687	147801258	chr1.trna23	571
1	147825439	147826010	chr1.trna24	571
1	147877287	147877860	chr1.trna25	573
1	148000555	148001128	chr1.trna26	573
1	148247865	148248438	chr1.trna27	573
1	148598064	148598637	chr1.trna98	573
1	148760106	148760679	chr1.trna97	573
1	149079114	149079685	chr1.trna28	571
1	149155578	149156149	chr1.trna96	571
1	149161378	149161949	chr1.trna95	571
1	149185875	149186446	chr1.trna29	571
1	149211698	149212271	chr1.trna94	573
1	149230320	149230893	chr1.trna93	573
1	149278535	149279107	chr1.trna92	572
1	149284253	149284826	chr1.trna91	573
1	149294416	149294986	chr1.trna90	570
1	149298305	149298877	chr1.trna89	572
1	149326022	149326595	chr1.trna88	573
1	149608359	149608932	chr1.trna30	573
1	149615367	149615940	chr1.trna87	573
1	149664105	149664677	chr1.trna86	572
1	149669807	149670380	chr1.trna85	573
1	149672660	149673221	chr1.trna84	561
1	149679965	149680525	chr1.trna83	560
1	149683838	149684411	chr1.trna82	573
1	149711548	149712121	chr1.trna81	573
1	150017132	150017702	chr1.trna80	570
1	153643476	153644047	chr1.trna31	571
1	159111151	159111724	chr1.trna79	573
1	161369240	161369812	chr1.trna78	572
1	161390411	161390983	chr1.trna32	572
1	161391633	161392204	chr1.trna77	571

1	161397617	161398190	chr1.trna76	573
1	161409711	161410282	chr1.trna75	571
1	161410365	161410936	chr1.trna74	571
1	161411073	161411655	chr1.trna33	582
1	161412844	161413414	chr1.trna34	570
1	161416768	161417339	chr1.trna73	571
1	161417125	161417696	chr1.trna72	571
1	161417783	161418354	chr1.trna71	571
1	161418491	161419073	chr1.trna35	582
1	161420217	161420787	chr1.trna36	570
1	161424148	161424719	chr1.trna70	571
1	161424506	161425077	chr1.trna69	571
1	161425164	161425735	chr1.trna68	571
1	161425872	161426454	chr1.trna37	582
1	161427648	161428218	chr1.trna38	570
1	161431559	161432130	chr1.trna67	571
1	161431916	161432487	chr1.trna66	571
1	161432574	161433145	chr1.trna65	571
1	161433282	161433864	chr1.trna39	582
1	161435008	161435578	chr1.trna40	570
1	161438939	161439510	chr1.trna64	571
1	161439297	161439868	chr1.trna63	571
1	161439955	161440526	chr1.trna62	571
1	161440663	161441245	chr1.trna41	582
1	161450106	161450676	chr1.trna42	570
1	161493387	161493957	chr1.trna61	570
1	161499882	161500464	chr1.trna60	582
1	161500653	161501224	chr1.trna43	571
1	161509781	161510354	chr1.trna44	573
1	161581486	161582069	chr1.trna59	583
1	161582258	161582829	chr1.trna45	571
1	161591215	161591788	chr1.trna46	573
1	165565900	165566472	chr1.trna47	572
1	167683712	167684283	chr1.trna48	571
1	167684475	167685046	chr1.trna58	571
1	180184026	180184598	chr1.trna57	572
1	204475405	204475977	chr1.trna49	572
1	204475908	204476480	chr1.trna56	572
1	205443021	205443593	chr1.trna50	572
1	222638097	222638669	chr1.trna51	572
1	238104124	238104689	chr1.trna52	565
1	238105612	238106183	chr1.trna53	571
1	249167804	249168409	chr1.trna54	605
1	249168197	249168768	chr1.trna55	571

2	11691583	11692143	chr2.trna1	560
2	27273400	27273988	chr2.trna2	588
2	27273832	27274404	chr2.trna3	572
2	30277318	30277890	chr2.trna4	572
2	43037426	43038018	chr2.trna5	592
2	45937191	45937763	chr2.trna26	572
2	70475873	70476443	chr2.trna25	570
2	85085626	85086198	chr2.trna6	572
2	117782259	117782830	chr2.trna24	571
2	117783761	117784326	chr2.trna23	565
2	121267430	121267985	chr2.trna22	555
2	131029661	131030232	chr2.trna21	571
2	131031149	131031713	chr2.trna20	564
2	131032774	131033343	chr2.trna19	569
2	131094451	131095022	chr2.trna18	571
2	132139892	132140461	chr2.trna7	569
2	132141402	132141966	chr2.trna8	564
2	132142883	132143454	chr2.trna9	571
2	156120039	156120604	chr2.trna10	565
2	157257031	157257602	chr2.trna11	571
2	157257409	157257979	chr2.trna17	570
2	203484384	203484949	chr2.trna12	565
2	204228881	204229451	chr2.trna16	570
2	219110299	219110891	chr2.trna13	592
2	219490895	219491449	chr2.trna14	554
2	224186065	224186637	chr2.trna15	572
3	13833636	13834203	chr3.trna1	567
3	15252723	15253298	chr3.trna2	575
3	17741146	17741711	chr3.trna9	565
3	45730241	45730813	chr3.trna8	572
3	106620628	106621199	chr3.trna7	571
3	131947694	131948265	chr3.trna6	571
3	131950392	131950963	chr3.trna5	571
3	169489768	169490340	chr3.trna3	572
3	184365845	184366415	chr3.trna4	570
4	39089849	39090410	chr4.trna5	561
4	40908493	40909065	chr4.trna4	572
4	124429755	124430326	chr4.trna3	571
4	156382152	156382717	chr4.trna1	565
4	156384728	156385302	chr4.trna2	574
5	26198289	26198861	chr5.trna25	572
5	58859569	58860151	chr5.trna1	582
5	131516222	131516781	chr5.trna2	559
5	141773738	141774309	chr5.trna24	571

5	151247824	151248395	chr5.trna23	571
5	151988346	151989021	chr5.trna3	675
5	168296009	168296577	chr5.trna4	568
5	180523820	180524392	chr5.trna5	572
5	180524224	180524805	chr5.trna22	581
5	180528590	180529171	chr5.trna6	581
5	180529003	180529575	chr5.trna21	572
5	180590904	180591476	chr5.trna7	572
5	180591322	180591886	chr5.trna20	564
5	180596360	180596932	chr5.trna8	572
5	180600400	180600972	chr5.trna9	572
5	180600794	180601375	chr5.trna19	581
5	180614451	180615032	chr5.trna10	581
5	180615166	180615738	chr5.trna18	572
5	180615604	180616175	chr5.trna17	571
5	180618437	180619008	chr5.trna16	571
5	180633618	180634189	chr5.trna11	571
5	180634505	180635077	chr5.trna12	572
5	180645020	180645592	chr5.trna15	572
5	180648729	180649301	chr5.trna14	572
5	180649145	180649717	chr5.trna13	572
6	18836152	18836723	chr6.trna1	571
6	26286504	26287075	chr6.trna2	571
6	26299655	26300227	chr6.trna3	572
6	26305471	26306048	chr6.trna176	577
6	26311174	26311745	chr6.trna175	571
6	26311725	26312296	chr6.trna174	571
6	26312574	26313155	chr6.trna173	581
6	26313102	26313673	chr6.trna172	571
6	26319080	26319651	chr6.trna171	571
6	26322796	26323368	chr6.trna4	572
6	26327567	26328148	chr6.trna5	581
6	26328118	26328690	chr6.trna6	572
6	26330279	26330850	chr6.trna170	571
6	26331422	26331993	chr6.trna169	571
6	26521186	26521768	chr6.trna7	582
6	26532895	26533468	chr6.trna168	573
6	26537476	26538048	chr6.trna8	572
6	26538032	26538604	chr6.trna9	572
6	26553481	26554052	chr6.trna10	571
6	26554100	26554673	chr6.trna11	573
6	26555248	26555819	chr6.trna12	571
6	26556524	26557096	chr6.trna13	572
6	26568836	26569426	chr6.trna14	590

6	26571842	26572414	chr6.trna167	572
6	26575548	26576137	chr6.trna15	589
6	26577082	26577670	chr6.trna16	588
6	26594852	26595440	chr6.trna17	588
6	26673340	26673912	chr6.trna18	572
6	26682465	26683037	chr6.trna19	572
6	26687235	26687807	chr6.trna20	572
6	26701462	26702034	chr6.trna21	572
6	26705356	26705928	chr6.trna22	572
6	26720971	26721544	chr6.trna166	573
6	26728006	26728578	chr6.trna23	572
6	26730487	26731059	chr6.trna24	572
6	26735324	26735896	chr6.trna165	572
6	26745005	26745578	chr6.trna164	573
6	26751668	26752240	chr6.trna25	572
6	26758300	26758872	chr6.trna163	572
6	26766194	26766766	chr6.trna26	572
6	26771040	26771612	chr6.trna162	572
6	26773521	26774093	chr6.trna161	572
6	26780582	26781155	chr6.trna27	573
6	26795756	26796328	chr6.trna160	572
6	26987875	26988468	chr6.trna28	593
6	27059271	27059842	chr6.trna29	571
6	27064835	27065416	chr6.trna30	581
6	27117772	27118344	chr6.trna31	572
6	27125656	27126227	chr6.trna32	571
6	27129800	27130373	chr6.trna33	573
6	27144744	27145317	chr6.trna159	573
6	27173617	27174189	chr6.trna158	572
6	27177378	27177959	chr6.trna34	581
6	27181373	27181945	chr6.trna157	572
6	27182702	27183274	chr6.trna35	572
6	27198084	27198666	chr6.trna156	582
6	27203038	27203610	chr6.trna36	572
6	27205100	27205673	chr6.trna155	573
6	27228460	27229034	chr6.trna37	574
6	27241489	27242062	chr6.trna38	573
6	27242740	27243313	chr6.trna154	573
6	27247799	27248371	chr6.trna153	572
6	27251614	27252187	chr6.trna39	573
6	27258155	27258727	chr6.trna40	572
6	27261421	27261994	chr6.trna41	573
6	27262962	27263533	chr6.trna42	571
6	27265525	27266106	chr6.trna43	581

6	27271318	27271889	chr6.trna152	571
6	27300514	27301085	chr6.trna151	571
6	27302519	27303091	chr6.trna150	572
6	27446341	27446922	chr6.trna44	581
6	27447203	27447774	chr6.trna45	571
6	27463343	27463924	chr6.trna46	581
6	27470568	27471149	chr6.trna47	581
6	27471273	27471844	chr6.trna48	571
6	27473357	27473938	chr6.trna149	581
6	27487058	27487629	chr6.trna49	571
6	27499737	27500318	chr6.trna50	581
6	27509304	27509885	chr6.trna148	581
6	27513218	27513799	chr6.trna51	581
6	27515281	27515852	chr6.trna147	571
6	27520942	27521523	chr6.trna146	581
6	27529713	27530299	chr6.trna52	586
6	27543596	27544168	chr6.trna53	572
6	27550986	27551557	chr6.trna145	571
6	27559343	27559915	chr6.trna144	572
6	27560350	27560921	chr6.trna143	571
6	27570098	27570704	chr6.trna142	606
6	27573167	27573774	chr6.trna141	607
6	27585885	27586458	chr6.trna54	573
6	27598950	27599543	chr6.trna55	593
6	27618457	27619029	chr6.trna140	572
6	27632295	27632870	chr6.trna56	575
6	27636112	27636685	chr6.trna57	573
6	27638094	27638666	chr6.trna139	572
6	27639979	27640560	chr6.trna138	581
6	27648635	27649207	chr6.trna137	572
6	27652224	27652797	chr6.trna136	573
6	27655717	27656290	chr6.trna58	573
6	27688648	27689230	chr6.trna135	582
6	27694223	27694796	chr6.trna59	573
6	27696077	27696649	chr6.trna134	572
6	27720929	27721501	chr6.trna133	572
6	27745414	27745985	chr6.trna60	571
6	27758885	27759456	chr6.trna132	571
6	27763390	27763961	chr6.trna131	571
6	27870021	27870592	chr6.trna130	571
6	27870436	27871006	chr6.trna129	570
6	28180565	28181146	chr6.trna61	581
6	28442079	28442652	chr6.trna128	573
6	28446150	28446731	chr6.trna127	581

6	28456520	28457093	chr6.trna126	573
6	28505117	28505710	chr6.trna62	593
6	28510641	28511213	chr6.trna125	572
6	28556906	28557477	chr6.trna63	571
6	28564867	28565448	chr6.trna124	581
6	28574683	28575254	chr6.trna64	571
6	28601609	28602180	chr6.trna123	571
6	28610972	28611543	chr6.trna65	571
6	28615734	28616307	chr6.trna122	573
6	28625764	28626335	chr6.trna121	571
6	28641363	28641934	chr6.trna120	571
6	28660737	28661310	chr6.trna119	573
6	28663459	28664030	chr6.trna118	571
6	28678116	28678687	chr6.trna66	571
6	28687231	28687802	chr6.trna67	571
6	28693545	28694118	chr6.trna68	573
6	28694605	28695177	chr6.trna117	572
6	28696842	28697413	chr6.trna69	571
6	28702956	28703527	chr6.trna116	571
6	28710479	28711051	chr6.trna115	572
6	28715271	28715843	chr6.trna70	572
6	28725891	28726462	chr6.trna114	571
6	28731124	28731697	chr6.trna113	573
6	28731914	28732483	chr6.trna71	569
6	28746294	28746865	chr6.trna112	571
6	28757297	28757868	chr6.trna111	571
6	28758249	28758821	chr6.trna110	572
6	28763491	28764062	chr6.trna109	571
6	28770327	28770897	chr6.trna108	570
6	28775360	28775932	chr6.trna107	572
6	28779599	28780170	chr6.trna106	571
6	28784762	28785333	chr6.trna105	571
6	28790843	28791416	chr6.trna104	573
6	28794940	28795511	chr6.trna103	571
6	28805971	28806542	chr6.trna102	571
6	28831212	28831783	chr6.trna101	571
6	28848915	28849487	chr6.trna72	572
6	28863750	28864355	chr6.trna100	605
6	28908580	28909184	chr6.trna73	604
6	28909128	28909699	chr6.trna99	571
6	28911149	28911730	chr6.trna98	581
6	28912102	28912674	chr6.trna74	572
6	28918556	28919128	chr6.trna75	572
6	28920792	28921364	chr6.trna97	572

6	28949199	28949771	chr6.trna96	572
6	28949726	28950297	chr6.trna76	571
6	28956529	28957110	chr6.trna77	581
6	58141627	58142199	chr6.trna95	572
6	58142339	58142911	chr6.trna94	572
6	58149004	58149577	chr6.trna78	573
6	58156200	58156772	chr6.trna93	572
6	58164378	58164950	chr6.trna92	572
6	58168242	58168814	chr6.trna91	572
6	58182429	58183001	chr6.trna90	572
6	58187194	58187766	chr6.trna89	572
6	58196373	58196945	chr6.trna88	572
6	69914128	69914710	chr6.trna79	582
6	79667763	79668332	chr6.trna87	569
6	126101143	126101714	chr6.trna86	571
6	142578526	142579096	chr6.trna80	570
6	144537434	144538016	chr6.trna81	582
6	145503609	145504180	chr6.trna82	571
6	152230027	152230590	chr6.trna83	563
6	156868796	156869370	chr6.trna85	574
6	158824398	158824970	chr6.trna84	572
7	57253730	57254301	chr7.trna32	571
7	63570292	63570857	chr7.trna1	565
7	66864297	66864868	chr7.trna31	571
7	68798379	68798945	chr7.trna2	566
7	96770638	96771202	chr7.trna30	564
7	99067057	99067628	chr7.trna3	571
7	128423254	128423825	chr7.trna4	571
7	139025196	139025768	chr7.trna5	572
7	141501623	141502188	chr7.trna6	565
7	141503107	141503678	chr7.trna7	571
7	149007031	149007602	chr7.trna8	571
7	149027970	149028541	chr7.trna9	571
7	149052516	149053087	chr7.trna29	571
7	149053505	149054065	chr7.trna28	560
7	149072600	149073171	chr7.trna27	571
7	149074351	149074922	chr7.trna26	571
7	149102905	149103476	chr7.trna25	571
7	149111979	149112550	chr7.trna24	571
7	149243381	149243952	chr7.trna10	571
7	149253552	149254121	chr7.trna11	569
7	149254883	149255455	chr7.trna12	572
7	149281566	149282137	chr7.trna13	571
7	149285914	149286485	chr7.trna23	571

7	149292055	149292626	chr7.trna22	571
7	149294796	149295367	chr7.trna14	571
7	149305217	149305788	chr7.trna15	571
7	149309906	149310477	chr7.trna21	571
7	149332528	149333099	chr7.trna16	571
7	149343796	149344367	chr7.trna20	571
7	149361665	149362236	chr7.trna17	571
7	149388022	149388593	chr7.trna19	571
7	149404510	149405081	chr7.trna18	571
8	59504548	59505118	chr8.trna1	570
8	66609282	66609869	chr8.trna11	587
8	67025352	67025944	chr8.trna2	592
8	67025973	67026561	chr8.trna3	588
8	67026174	67026746	chr8.trna4	572
8	89144986	89145559	chr8.trna10	573
8	96281635	96282216	chr8.trna9	581
8	99153212	99153780	chr8.trna5	568
8	111946528	111947093	chr8.trna6	565
8	124169220	124169792	chr8.trna8	572
8	134767760	134768325	chr8.trna7	565
9	5096337	5096902	chr9.trna10	565
9	14433688	14434259	chr9.trna9	571
9	19403746	19404320	chr9.trna1	574
9	77517740	77518311	chr9.trna8	571
9	83179311	83179874	chr9.trna2	563
9	86861165	86861724	chr9.trna3	559
9	95301230	95301795	chr9.trna4	565
9	112960553	112961125	chr9.trna5	572
9	123117103	123117685	chr9.trna7	582
9	131102105	131102695	chr9.trna6	590
10	5895424	5895996	chr10.trna7	572
10	20036368	20036924	chr10.trna6	556
10	22518188	22518761	chr10.trna5	573
10	22852329	22852901	chr10.trna1	572
10	34591166	34591733	chr10.trna4	567
10	69524011	69524592	chr10.trna2	581
10	71352447	71353012	chr10.trna3	565
11	9296540	9297113	chr11.trna1	573
11	20221546	20222101	chr11.trna21	555
11	50233629	50234200	chr11.trna20	571
11	51359652	51360220	chr11.trna2	568
11	59317852	59318424	chr11.trna19	572
11	59318210	59318782	chr11.trna18	572
11	59318517	59319102	chr11.trna3	585

11	59318978	59319560	chr11.trna4	582
11	59323652	59324224	chr11.trna5	572
11	59324720	59325292	chr11.trna17	572
11	59327558	59328130	chr11.trna16	572
11	59333603	59334175	chr11.trna15	572
11	66115341	66115922	chr11.trna6	581
11	75214277	75214849	chr11.trna14	572
11	75946307	75946878	chr11.trna7	571
11	75946619	75947190	chr11.trna13	571
11	103274716	103275285	chr11.trna8	569
11	103276327	103276892	chr11.trna9	565
11	109035791	109036368	chr11.trna10	577
11	113432745	113433328	chr11.trna12	583
11	122430405	122430977	chr11.trna11	572
12	27843056	27843628	chr12.trna1	572
12	50210940	50211514	chr12.trna15	574
12	56583898	56584479	chr12.trna2	581
12	70220508	70221077	chr12.trna14	569
12	74850932	74851504	chr12.trna3	572
12	96429549	96430120	chr12.trna4	571
12	98897031	98897602	chr12.trna5	571
12	98897780	98898351	chr12.trna6	571
12	122860674	122861244	chr12.trna7	570
12	125406051	125406622	chr12.trna13	571
12	125411641	125412212	chr12.trna12	571
12	125412139	125412711	chr12.trna11	572
12	125423943	125424514	chr12.trna10	571
12	125424262	125424833	chr12.trna8	571
12	129715948	129716522	chr12.trna9	574
13	31247851	31248424	chr13.trna5	573
13	41634624	41635195	chr13.trna4	571
13	42029811	42030382	chr13.trna3	571
13	45491812	45492383	chr13.trna2	571
13	95201654	95202226	chr13.trna1	572
14	21077245	21077816	chr14.trna24	571
14	21078041	21078622	chr14.trna1	581
14	21081310	21081881	chr14.trna23	571
14	21081699	21082271	chr14.trna22	572
14	21093279	21093860	chr14.trna2	581
14	21099069	21099641	chr14.trna21	572
14	21100915	21101486	chr14.trna3	571
14	21121008	21121601	chr14.trna20	593
14	21125373	21125966	chr14.trna19	593
14	21127867	21128460	chr14.trna18	593

14	21131101	21131694	chr14.trna17	593
14	21144928	21145509	chr14.trna16	581
14	21149599	21150171	chr14.trna4	572
14	21151182	21151770	chr14.trna5	588
14	21151925	21152496	chr14.trna6	571
14	23398660	23399232	chr14.trna7	572
14	32236567	32237135	chr14.trna15	568
14	32953768	32954333	chr14.trna8	565
14	58706363	58706935	chr14.trna14	572
14	73429429	73430000	chr14.trna9	571
14	74055285	74055846	chr14.trna13	561
14	89445192	89445764	chr14.trna10	572
14	91726312	91726884	chr14.trna11	572
14	102783179	102783752	chr14.trna12	573
15	26327131	26327702	chr15.trna12	571
15	40885773	40886354	chr15.trna11	581
15	45490554	45491125	chr15.trna10	571
15	45492361	45492932	chr15.trna9	571
15	45493099	45493670	chr15.trna1	571
15	66161150	66161721	chr15.trna8	571
15	76674515	76675088	chr15.trna7	573
15	79152654	79153226	chr15.trna2	572
15	80036747	80037319	chr15.trna3	572
15	89878054	89878626	chr15.trna4	572
15	92254052	92254624	chr15.trna6	572
15	96327629	96328188	chr15.trna5	559
16	686486	687056	chr16.trna32	570
16	2977412	2977984	chr16.trna1	572
16	3200425	3200997	chr16.trna2	572
16	3202651	3203223	chr16.trna3	572
16	3207156	3207728	chr16.trna31	572
16	3208673	3209244	chr16.trna4	571
16	3210136	3210730	chr16.trna5	594
16	3214689	3215261	chr16.trna6	572
16	3221799	3222370	chr16.trna7	571
16	3225442	3226014	chr16.trna8	572
16	3230305	3230877	chr16.trna30	572
16	3232385	3232956	chr16.trna29	571
16	3233883	3234454	chr16.trna28	571
16	3237844	3238415	chr16.trna9	571
16	3239384	3239955	chr16.trna10	571
16	3241251	3241823	chr16.trna11	572
16	3241739	3242310	chr16.trna12	571
16	3243668	3244240	chr16.trna13	572

16	3245903	3246475	chr16.trna14	572
16	3419563	3420134	chr16.trna15	571
16	14379500	14380071	chr16.trna16	571
16	22206782	22207363	chr16.trna27	581
16	22308211	22308792	chr16.trna17	581
16	57333613	57334195	chr16.trna18	582
16	57334142	57334724	chr16.trna26	582
16	70811864	70812434	chr16.trna25	570
16	70812692	70813262	chr16.trna24	570
16	70822347	70822917	chr16.trna19	570
16	70823160	70823730	chr16.trna20	570
16	71460146	71460718	chr16.trna21	572
16	73511966	73512538	chr16.trna23	572
16	87417378	87417950	chr16.trna22	572
17	8022223	8022795	chr17.trna1	572
17	8022820	8023391	chr17.trna2	571
17	8023382	8023963	chr17.trna42	581
17	8023993	8024580	chr17.trna3	587
17	8028814	8029384	chr17.trna4	570
17	8041949	8042530	chr17.trna41	581
17	8042520	8043093	chr17.trna40	573
17	8089426	8089997	chr17.trna5	571
17	8089934	8090515	chr17.trna6	581
17	8090228	8090801	chr17.trna7	573
17	8090661	8091234	chr17.trna8	573
17	8123937	8124508	chr17.trna39	571
17	8124616	8125187	chr17.trna9	571
17	8125306	8125877	chr17.trna38	571
17	8125901	8126472	chr17.trna37	571
17	8129303	8129876	chr17.trna36	573
17	8129678	8130259	chr17.trna35	581
17	8130059	8130632	chr17.trna34	573
17	19411244	19411815	chr17.trna10	571
17	19508430	19508995	chr17.trna33	565
17	19763925	19764495	chr17.trna11	570
17	22026327	22026882	chr17.trna32	555
17	22027929	22028497	chr17.trna31	568
17	29876843	29877414	chr17.trna12	571
17	36907784	36908357	chr17.trna30	573
17	36989725	36990296	chr17.trna29	571
17	37017687	37018258	chr17.trna28	571
17	37023648	37024219	chr17.trna13	571
17	37025295	37025866	chr17.trna27	571
17	37309737	37310308	chr17.trna26	571

17	37310494	37311065	chr17.trna25	571
17	38238683	38239255	chr17.trna24	572
17	38273303	38273876	chr17.trna23	573
17	47269640	47270211	chr17.trna14	571
17	58863343	58863913	chr17.trna15	570
17	62526668	62527241	chr17.trna22	573
17	66015763	66016335	chr17.trna21	572
17	66390707	66391281	chr17.trna20	574
17	73029751	73030323	chr17.trna16	572
17	73030276	73030848	chr17.trna19	572
17	73030958	73031530	chr17.trna17	572
17	80452347	80452918	chr17.trna18	571
18	43669021	43669593	chr18.trna1	572
19	1383111	1383683	chr19.trna14	572
19	1383312	1383885	chr19.trna1	573
19	4723832	4724403	chr19.trna2	571
19	4724397	4724969	chr19.trna13	572
19	12299126	12299699	chr19.trna3	573
19	32486465	32487032	chr19.trna4	567
19	33667713	33668286	chr19.trna5	573
19	36066500	36067072	chr19.trna6	572
19	36111210	36111780	chr19.trna12	570
19	39902558	39903150	chr19.trna11	592
19	41747892	41748464	chr19.trna10	572
19	45981610	45982195	chr19.trna9	585
19	50037683	50038255	chr19.trna8	572
19	52425148	52425718	chr19.trna7	570
20	48952092	48952673	chr20.trna1	581
21	10492722	10493287	chr21.trna2	565
21	18826857	18827427	chr21.trna1	570
22	44546287	44546872	chr22.trna1	585
X	3756168	3756741	chrX.trna10	573
X	3756582	3757155	chrX.trna9	573
X	3794592	3795165	chrX.trna8	573
X	3795006	3795579	chrX.trna7	573
X	3833021	3833594	chrX.trna6	573
X	3833435	3834008	chrX.trna5	573
X	18692779	18693351	chrX.trna4	572
X	24619704	24620272	chrX.trna3	568
X	55206416	55206987	chrX.trna1	571
X	55207505	55208079	chrX.trna2	574

[a]Start and stop positions were obtained from GtRNAdb and the University of California Santa Cruz (UCSC) genome browser using the February 2009 CRCh37/hg19 genome build.

Table S2. tDNA loci not identified in our analysis.

tRNA Gene	Chromosome	Start	Stop	Length
tRNA-Asp-GTC-4-1	9	77517990	77518061	72
tRNA-Leu-CAG-1-1	1	161411323	161411405	83
tRNA-Cys-ACA-1-1	5	151988596	151988771	176
tRNA-Val-TAC-chr17-24	17	38238933	38239005	73
tRNA-Lys-TTT-10-1	19	41748142	41748214	73

Table S3. tRNA variants predicted to change functionality based on Infernal score.

tRNA Gene	Variant	Reference Infernal Score	Variant Infernal Score	Predicted Functional Change to:
tRNA-Und-NNN-3-1	chr1:161390723:C/T	36.3	42.7	Not Pseudo
tRNA-Ser-ACT-1-1	chr6:27261700:C/G	47	56.9	Not Pseudo
tRNA-Phe-GAA-10-1	chr1:149672939:C/T<br>chr1:149672936:A/G	18.9	32.2	Not Pseudo
tRNA-Ser-ACT-1-1	chr6:27261700:C/G<br>chr6:27261706:C/T	47	56.9	Not Pseudo
tRNA-Cys-GCA-24-1	chr17:36990037:C/-	47.6	41.4	Pseudo
tRNA-Thr-CGT-6-1	chr6:27271634:T/C<br>chr6:27271590:A/G	37	30.4	Pseudo
tRNA-Gly-CCC-5-1	chr1:17053826:C/T	56.3	49.6	Pseudo
tRNA-Und-NNN-5-1	chr17:66390978:C/T	33.6	25.9	Pseudo
tRNA-Gln-TTG-chr8-10	chr8:89145307:A/G	21.5	15.8	Pseudo
tRNA-Cys-GCA-24-1	chr17:36990039:C/-	47.6	41.4	Pseudo
